# Alternative and Augmentative Communication Technologies for Supporting Adults With Mild Intellectual Disabilities During Clinical Consultations: Scoping Review

**DOI:** 10.2196/19925

**Published:** 2021-06-09

**Authors:** Ryan Colin Gibson, Matt-Mouley Bouamrane, Mark D Dunlop

**Affiliations:** 1 Department of Computer and Information Sciences University of Strathclyde Glasgow United Kingdom; 2 Usher Institute of Population Health Sciences and Informatics University of Edinburgh Edinburgh United Kingdom

**Keywords:** intellectual disabilities, health care, communication, alternative and augmentative communication, communication modalities, mobile applications, patient passports, Talking Mats, health assessment booklets

## Abstract

**Background:**

People with intellectual disabilities (IDs) face significant communication barriers when accessing health care services; they find it difficult to identify and describe conditions clearly enough to support practitioners in making an accurate diagnosis. In addition, medical professionals generally have little knowledge and understanding of the needs of people with ID, which may result in the use of consultation techniques that do not cater to their patients’ skills.

**Objective:**

This review aims to identify and synthesize the literature on alternative and augmentative communication technologies that are used to support adults with mild ID during the exchange of information with medical practitioners.

**Methods:**

We performed a scoping review of studies published in English that describe the technologies that are used to promote communication with patients with mild ID during medical consultations. The databases searched were PubMed, ACM Digital Library, and Google Scholar. A qualitative framework-based approach was used to synthesize the data and discern key recurring themes across the identified literature.

**Results:**

Of the 1557 articles screened, 15 (0.96%) met our inclusion criteria. The bulk of the communication aids used focused on low-tech solutions, including patient passports, note-based prompts, Talking Mats, health diaries, and easy-read information sheets. Their influence on current practice ranged from advancing medical professionals’ knowledge of the health and communication needs of people with ID to increasing interagency collaboration, patient advocacy skills, and health promotion activities. The major barriers to the implementation of low-tech aids were a lack of portability and increased maintenance efforts. Only 3 studies explored the use of mobile apps to promote communication. Their findings indicated that high-tech solutions offer greater customization with regard to the accessibility and health care needs of people with ID.

**Conclusions:**

Alternative and augmentative communication technologies have the potential to increase the quality of care provided to patients with mild ID; however, little work has been carried out in this area. Greater emphasis must be placed on (high-tech) two-way communication aids that empower patients to become involved in decisions regarding their care. Quantitative evaluation methods should be used to discern the true benefits of such aids, and researchers should describe their study protocols in depth to promote replication and generalizability.

## Introduction

### Background

People with intellectual disabilities (IDs) are consistently subjected to health inequalities [1-5], which significantly affect the length and standard of their lives [4,6,7]. Heslop et al [4] demonstrated this in 2013 while studying the deaths of 247 patients with ID in southwest England; of those deaths, 103 (41.7%) were classified as unexpected or premature, with 68 (27.5%) directly attributable to low-quality care. In addition, the patients suffered from an average of five long-term or treatable conditions during the period leading up to their deaths [4], several of which were straightforward to diagnose and cure, for example, constipation in 37% of cases and pressure sores in 34% of cases.

Previous literature has suggested that many of the inequalities experienced by patients with ID are preventable, particularly the breakdown in communication with health professionals [1-3,5]. To overcome such communication barriers and therefore provide improved person-centered care, practitioners are encouraged to use national [8,9] and international [10] guidelines. Much of this advice centers on the implementation of reasonable adjustments that cater to the patient’s individual needs. These adjustments include aspects such as using the patient’s preferred method of communication, avoiding the use of medical jargon to cater to their reduced vocabulary and cognitive abilities, and ensuring that patients understand the information conveyed to overcome impairments in their receptive skills [8-10]. In addition, the practitioner should consider and be vigilant for gestures that emphasize the information being exchanged, assign additional time to the consultation to allow the patient to deliberate what has been said, and provide information in advance of the appointment to allow the patient to prepare adequately [8-10].

However, medical professionals frequently report that they are undertrained on the health and accessibility needs of people with ID [11-13] and therefore lack the confidence and skills to implement the proposed guidelines. In this context, innovative practices have been introduced to improve the standard of care administered [1,2]. The bulk of these innovative practices attempt to mitigate the gaps in knowledge held by staff via the establishment of patient-focused training sessions and the increased availability of ID information resources. In addition, health care organizations have changed their pathways to include targeted health check programs that assist in diagnosing common conditions experienced by people with ID. Multidisciplinary teams of health professionals have also been formed to support this process, including the specialized skills possessed by ID nurses [1,2].

Nevertheless, medical staff are currently overworked [14,15], meaning they have limited opportunities to seek additional training. This, combined with the decline in the number of specialized professionals such as ID nurses [16], suggests that other forms of support must be explored to promote communication between practitioners and patients with ID.

### Objective

Technology has the potential to provide such support as it has been shown to enhance the lives of people with cognitive, intellectual, or physical disabilities [17-19]; however, little is known about its use in the clinical context for people with ID. Consequently, we conducted a scoping review to synthesize the literature on the use of communication technologies to support people with mild ID during clinical consultations. The results of our review are presented in the form of four themes that were developed using thematic framework analysis.

## Methods

### ID Definition

Throughout this paper, we refer to the term *intellectual disabilities* by using the World Health Organization definition: “a significantly reduced ability to understand new or complex information and to learn and apply new skills (impaired intelligence). This results in a reduced ability to cope independently (impaired social functioning) and begins before adulthood, with a lasting effect on development” [20]. There are several manifestations of ID, with their impact on an individual’s social and cognitive functioning ranging from mild to severe [21]. This review focuses on people with mild ID who, in general, live independent lives but may require support to complete complex processes such as understanding medical conditions. We hypothesize that this population is more likely to benefit from health-related interventions, such as digital communication aids, as people with severe ID tend to seek support during basic tasks, meaning they are unlikely to use such technologies autonomously or be in charge of their own health care.

### Aim

This review aims to identify and synthesize a range of technologies and modalities used to promote communication between patients with mild ID and health professionals. Consequently, the research question underpinning this review is, “What technologies are being used to support adults with mild ID to communicate more effectively with medical practitioners?**”**

In addition to these research questions, the scoping review has the following objectives:

- Subobjective 1: determine how the identified aids were being used by patients with mild ID and medical professionals
- Subobjective 2: determine how the benefits of the aids were evaluated

Our work differs from that of Chinn [22], as its focus is on the technologies being used by patients with mild ID instead of other forms of support such as health-related training courses.

### Research Methodology

Arksey and O’Malley [23] presented four common scenarios where scoping reviews are an appropriate methodology to use, two of which align with our research objectives: (1) examining the extent, range, and nature of research activity within a domain and (2) identifying research gaps within the existing literature. As such, the framework of Arksey and O’Malley [23] was used to rapidly map the key concepts within our target domain, which consisted of the following 5 flexible steps:

- Research question formulation (*Aim* section)
- Identification of relevant studies (*Search Strategy* section)
- Study selection (*Inclusion Criteria* and *Study Selection* sections)
- Charting the data (*Data Charting* section)
- Collating, summarizing, and reporting the results (*Analysis* section)

### Search Strategy

To conduct a holistic search that included technological, sociotechnical, and disability-focused communication studies, 3 databases were queried (PubMed, ACM Digital Library, and Google Scholar) using the terms shown in Textbox 1. These phrases were based on Medical Subject Headings relating to communication, ID, and clinical consultations in conjunction with a variety of alternative and augmentative communication (AAC) technologies. In all, 15 queries were carried out (Textbox 1), resulting in the identification of 1737 articles published before November 2019: 747 from PubMed, 140 from ACM, and 850 from Google Scholar. Separate queries were used per database because of their differing scopes. For example, it was not appropriate to search for Talking Mats or patient passports in the ACM database as the articles returned primarily focused on high-tech interventions such as mobile apps.

PubMed was selected because of its focus on medical studies, including those that discuss the implementation of interventions. Each of the unique articles retrieved from PubMed had their titles and abstracts screened by RCG against the inclusion and exclusion criteria described in the following subsection. Potentially relevant articles were then read in their entirety to identify those that adhered to the selection criteria, with more obscure articles being analyzed by MMB before their inclusion or omission. The areas of conflict between the first and second authors were resolved by MDD. Searches across the 3 databases resulted in 5 articles that were reviewed by RCG and MMB, of which 2 were also reviewed by MDD.

ACM was identified because of its focus on technology, particularly articles centering on the development of AAC aids. In addition, the literature returned by ACM does not overlap with that identified by PubMed, which increases the comprehensiveness of the search. Relevant articles were chosen using the same process as described above.

Finally, Google Scholar was selected as it is often used to supplement evidence searches by returning relevant articles cataloged in databases beyond those originally queried [24]. Researchers often limit their Google Scholar queries to the first 50 to 100 articles [24] because as a ranked retrieval system, the relevance of the literature diminishes as the search progresses. However, we increased this number to 200, based on the following procedure: the search results for query 1 (Textbox 1) were split into groups of 50. The first batch of 50 was then screened (using the same process as the previous databases), with the investigators moving on to the next batch only if a potentially relevant article was identified via its abstract; otherwise, the search was terminated. This procedure was repeated for queries 2 to 5, with the highest batch number being used as a limit for all Google Scholar searches. To elaborate, a relevant article was identified in the third batch of the second query, meaning the first 200 results of the other queries were scrutinized where possible. Nevertheless, it is important to note that some of the searches returned less than 200 articles, meaning all were scrutinized as the N size fell below the defined limit.

**Figure 1:**
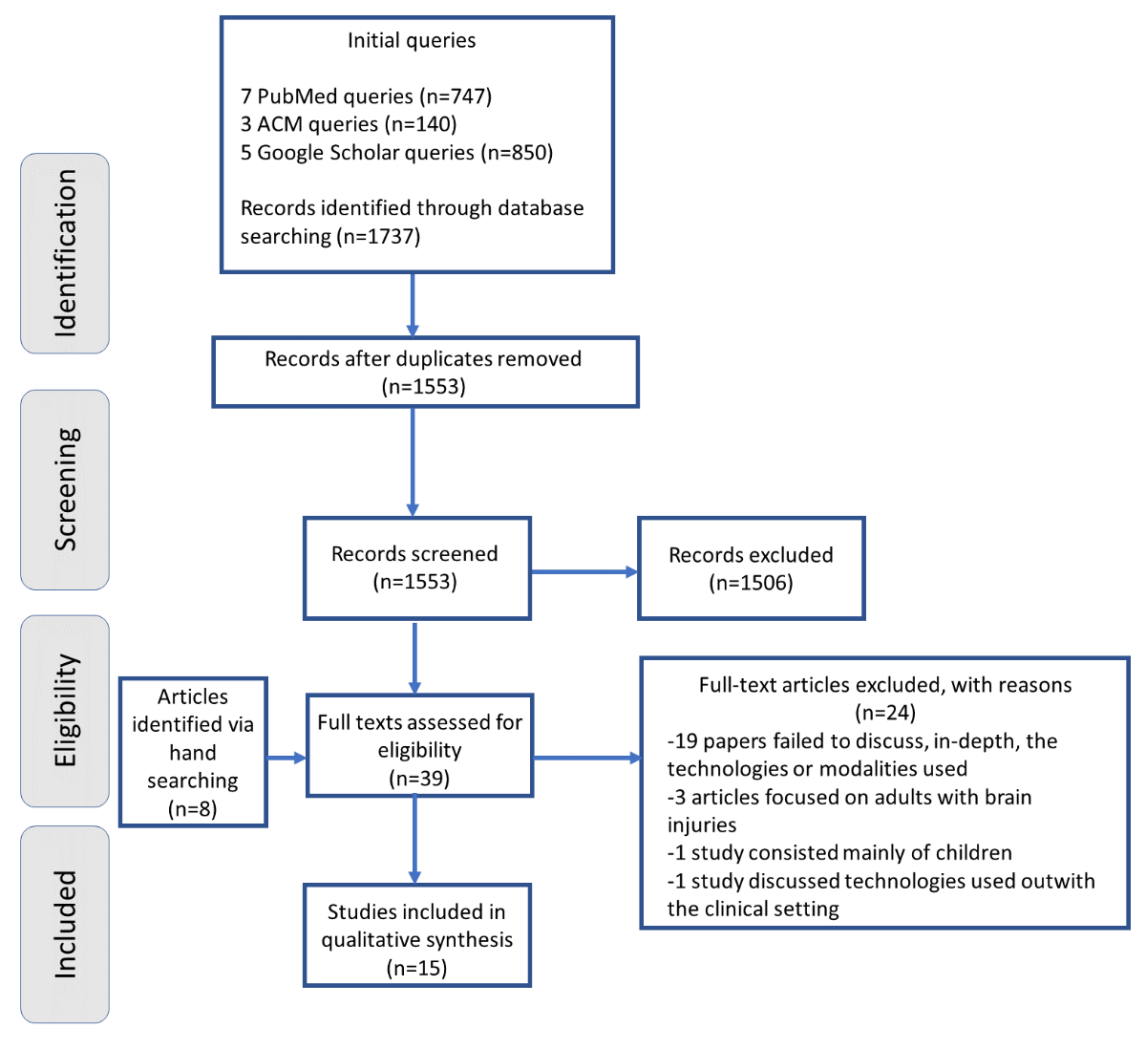
PRISMA (Preferred Reporting Items for Systematic Reviews and Meta-Analyses) flowchart of this scoping review.

Figure 1 contains a PRISMA (Preferred Reporting Items for Systematic Reviews and Meta-Analyses) flow diagram [25] detailing the steps involved in identifying relevant articles. These articles were then subjected to a qualitative framework-based analysis to synthesize the results and determine key recurrent themes (*Analysis* section).

**Search queries and search terms.**

**PubMed**

- Query 1
  ((“intellectual disability”[MeSH Terms] OR (“intellectual”[All Fields] AND “disability”[All Fields]) OR “intellectual disability”[All Fields]) AND (“communication”[MeSH Terms] OR “communication”[All Fields])) AND (“referral and consultation”[MeSH Terms] OR (“referral”[All Fields] AND “consultation”[All Fields]) OR “referral and consultation”[All Fields] OR “consultations”[All Fields])
- Query 2
  ((Alternative[All Fields] AND Augmentative[All Fields] AND (“communication”[MeSH Terms] OR “communication”[All Fields])) AND (“learning disorders”[MeSH Terms] OR (“learning”[All Fields] AND “disorders”[All Fields]) OR “learning disorders”[All Fields] OR (“learning”[All Fields] AND “disabilities”[All Fields]) OR “learning disabilities”[All Fields])) AND clinical[All Fields]
- Query 3
  ((“speech”[MeSH Terms] OR “speech”[All Fields] OR “talking”[All Fields]) AND “mats”[All Fields])) AND clinical[All Fields]
- Query 4
  (alternative[All Fields] AND augmentative[All Fields] AND (“communication”[MeSH Terms] OR “communication”[All Fields])) AND clinical[All Fields]
- Query 5
  ((“communication”[MeSH Terms] OR “communication”[All Fields] OR (“personal”[All Fields] AND “communication”[All Fields]) OR “personal communication”[All Fields]) AND passports[All Fields]) AND clinical[All Fields]
- Query 6
  (pictures[All Fields] OR images[All Fields] OR graphics[All Fields]) AND clinical[All Fields] AND ((intellectual[All Fields] OR (“learning”[MeSH Terms] OR “learning”[All Fields])) AND disabilities[All Fields])
- Query 7
  ((“communication”[MeSH Terms] OR “communication”[All Fields]) AND (((“learning”[MeSH Terms] OR “learning”[All Fields]) OR intellectual[All Fields]) AND disabilities[All Fields])) AND clinical[All Fields]

**ACM Digital Library**

- Query 1
  ((“intellectual” AND disability”) AND communication) AND consultations
- Query 2
  (“Alternative” AND “Augmentative” AND “Communication”) AND (“Learning” AND “Disabilities”) AND “clinical”
- Query 3
  (pictures images graphics “clinical” disabilities) AND recordAbstract:(+intellectual +learning)

**Google Scholar**

- Query 1
  ((“intellectual” AND “disability”) AND “communication”) AND “consultations”
- Query 2
  ((“Alternative” AND “Augmentative” AND “communication”)) AND “learning disabilities”) AND “clinical”)
- Query 3
  (“Talking” AND “Mats”) AND (“learning” AND “disabilities”) AND “clinical”
- Query 4
  (“personal” AND “communication” AND “passports”) AND (“learning” AND “disabilities”) AND “clinical”
- Query 5
  allintitle: “clinical” AND “disabilities” AND “pictures” OR “images” OR “graphics” OR “intellectual” OR “learning”

### Inclusion Criteria

The review was restricted to literature that discussed the use of technology to promote communication between patients with mild IDs and health professionals. Textbox 2 describes the inclusion criteria used to screen relevant articles based on the PICOS (participants, intervention, comparison, outcomes, and study) search tool [26].

Articles may also have been excluded if they were deemed to be of low quality by any research team member. This was assessed using the following three characteristics based on the aspects identified by Alborz et al [1]: (1) clarity of research questions or goals; (2) appropriateness of the methods employed in relation to the research questions; (3) and consideration of study limitations.

N size is often used as a proxy for the quality of a study; however, it was not considered appropriate for article exclusion because of our interest in the development of technologies and their implementation.

**Inclusion criteria for relevant articles.**

**Participants**

- Adults aged 18 years or older with mild intellectual disabilities and health professionals; studies were also included where little information on the participants’ intellectual disability was provided.
- We used the World Health Organization’s definition of intellectual disability [20], which therefore rules out conditions linked to cognitive decline because of aging or other neurological disorders acquired later in life, for example, dementia. Participants with physical disabilities (eg, cerebral palsy) and no accompanying cognitive impairments were also excluded.

**Interventions**

- A range of communication modalities or technologies used to promote the exchange of information between patients with mild intellectual disability and health professionals during clinical consultations. This, therefore, excludes clinical studies with no focus on communication and evaluation of aids used to manage a specific condition. To be considered relevant, articles had to describe the components that comprised the aid. For example, it was not enough to state that a patient passport was used; rather, the characteristics included in the passport also had to be described. As such, the elements that influenced practice could be identified.

**Comparator**

- The review was not limited to comparator studies.

**Outcomes**

- Qualitative and quantitative data reporting the effects of communication aids and modalities on clinical consultations involving adult patients with mild intellectual disability.

**Study type**

- Primary studies only were considered relevant in this review.

### Study Selection

As shown in Figure 1, a total of 15 articles met our inclusion criteria. Of the initial 1553 articles that had their abstracts screened, 1514 (97.49%) were immediately excluded from the review. Consequently, 39 were read in their entirety, of which 15 (38%) were deemed appropriate for inclusion in the review. A total of 20 articles were excluded because they did not fit our intervention inclusion criteria, and a further 4 were excluded because they failed to meet our participants’ inclusion criteria. No articles were excluded based on quality.

### Data Charting

RCG and MMB jointly developed a data-charting form to extract relevant information from the identified studies. The characteristics within this form were similar to those proposed by Arksey and O’Malley [23] and included authors year of publication, study location, study aim, intervention, study design, populations, and key results. The same authors independently charted the data and discussed their conclusions with MDD on hand to resolve any discrepancies. A summary of the charted data is provided in Multimedia Appendix 1 [27-41].

### Analysis

A deductive, framework-based analysis [42] was used to synthesize charted data. RCG developed an initial thematic model capable of answering the research objectives proposed by using the communication barriers or facilitators discussed in other reviews [43,44]. This model was then discussed by the coauthors and agreed upon by consensus. RCG then applied the framework to a subset of the articles (consisting of 1 study per distinct AAC aid identified) and subsequently extended it where necessary, under the guidance of Gale et al [42], to include important aspects of the data that did not immediately adhere to the original concepts. To limit bias, Gale et al [42] also suggested that researchers reach a consensus on the coding applied to at least the first few transcripts. As such, MMB proceeded to review the tagged data, with any discrepancies in the applied framework being resolved by MDD. The remaining articles were then analyzed by RCG using this framework, with additional subthemes being created as required. MB and MDD were consulted on the creation of new tags to ensure that they were necessary and did not align with the other concepts. The final revised thematic framework can be found on the the University of Strathclyde website [45]; a summary of the themes is provided in Textbox 3.

**Thematic framework.**

**Communication barriers and facilitators**

- This theme addresses the various practices that have an adverse or positive impact on information exchanges between medical professionals and patients with mild intellectual disability, covering aspects such as organizational procedures, fragmentation of care, education and training opportunities, and person-centered care.

**Technological aids**

- This theme identifies the various forms of communication aids used by patients and practitioners during clinical consultations and has been split into two primary subthemes: paper-based technologies and more complex digital technologies. An overview of the features included within each aid is provided.

**Communication modalities**

- This theme introduces the communication modalities employed throughout the aids, including the benefits and drawbacks of each. It also highlights the need for technologies to be adaptive because of the wide range of skills and needs experienced by people with intellectual disability, meaning a one-size-fits-all approach is unsuitable.

**Evaluation and impact**

- This theme discusses the various qualitative and quantitative methods used within the identified studies. It also introduces the perceived impact of the communication aids under scrutiny.

## Results

In this section, we first present the general characteristics of the identified studies (publication and participants) before discussing the results of the framework-based thematic analysis. An in-depth description of the selected studies may be found in Multimedia Appendix 1, with a short summary provided in Table 1.

**Table 1:** Short overview of the identified studies.

Study (Author [year]; assessment tool)	Complexity		Modality			Participants		Evaluation	
	High-tech	Low-tech	Text	Imagery	Speech	Mainly people with ID^a^	Mainly other populations	Qualitative	Quantitative
Jones and Kerr [27] (1997); paper-based checklist		✓^b^	✓			✓			✓
Dodd and Brunker [28] (1999); image cards and training session		✓	✓	✓		✓		✓	
Lennox et al [29] (2001); CHAP^c^		✓	✓			✓		✓	
Lennox et al [30] (2004); Ask It Health Diary		✓	✓	✓			✓	✓	
Bell and Cameron [31] (2008); Talking Mats		✓	✓	✓		✓		✓	
Lennox et al [32] (2010); CHAP and Ask It		✓	✓	✓		✓			✓
Turk et al [33] (2010); hand-held health record		✓	✓			✓			✓
Brodrick et al [34] (2011); patient passport		✓	✓				✓	✓	
Bell [35] (2012); patient passport		✓	✓			✓		✓	
Heifetz and Lunsky [36] (2018); patient passport		✓	✓				✓	✓	
Gibson et al [37] (2018); tablet app	✓		✓	✓	✓		✓	✓	
Gibson et al [38] (2019); tablet app	✓		✓	✓	✓		✓	✓	
Gibson et al [39] (2019); tablet app	✓		✓	✓	✓		✓	✓	
Raemy and Pignon [40] (2019); patient passport		✓	✓				✓	✓	
Chinn [41] (2019); easy-read health information		✓	✓	✓		✓		✓	

^a^ID: intellectual disability.

^b^Checkmark indicates the presence of that characteristic within the study.

^c^CHAP: Comprehensive Health Assessment Program.

### Overview of the Studies Selected

#### Publication

Of the 15 articles that met our inclusion criteria, 9 (60%) were retrieved from PubMed [27,29,30,34,36,38-41], 5 (33%) from Google Scholar [28,31-33,35], and 1 (7%) from ACM [37]. In total, 13% (2/15) were published in the 1990s [27,28], 20% (3/15) were published in the 2000s [29-31], and 67% (10/15) in the 2010s [32-41]. This finding highlights a substantial increase in the number of studies published on the focus of our review since the turn of the millennium and is in line with the heightened awareness of issues relating to the accessibility of services for people with ID [46,47]. However, despite such an increase, the study of Hemsley and Balandin [43] on the quality of communication between medical professionals and patients with severe communication disabilities concluded that the use of AAC in this context remains limited. Environmental barriers were cited as negatively affecting the implementation of AAC technologies, as was the knowledge of staff who find it difficult to adapt to technologies brought in externally by patients [43].

Furthermore, all studies identified during the data collection phase were carried out in countries that are members of the Organization for Economic Cooperation and Development (OECD), with most centering on the health care infrastructure of the United Kingdom [27,28,31,33-35,37-39,41] and Australia [29,30,32]. As such, the generalizability of the findings may be limited, particularly regarding the impact of AAC technologies on patients with ID from non-OECD nations.

#### Participants

##### Participants Involved in the Design and Development of an Intervention

In total, 6 of the articles described the design and development of an intervention to promote communication between adult patients with mild ID and health professionals [30,34,37-40]. Surprisingly, target stakeholders were not heavily involved in the design process (despite increasing expectations of the use of co-design methodologies [48]), with investigators largely deferring to the views of other populations. For example, Lennox et al [30] relied upon an advisory group (consisting of 2 individuals with ID, 2 support workers, 2 parents, 2 advocacy organization representatives, and an occupational therapist) to develop a health diary for persons with ID. Their initial designs were then scrutinized, before implementation, by 101 people across 15 focus groups, yet health professionals (1 general practitioner [GP] and 2 psychologists) and patients with ID (8 persons) were underrepresented during this process.

Both Brodrick et al [34] and Raemy and Paignon [40] also followed the approach of using a multidisciplinary team to develop their respective interventions—a 1-page patient passport and an emergency admission sheet. However, the authors failed to report the exact demographics of the members involved, meaning it was difficult to discern the influence people with ID had on the design process. This was particularly true in the study of Brodrick et al [34], where it was unclear whether the ID population had any input on the intervention design.

Finally, Gibson et al [37-39] used a variety of experts in ID (researchers, support workers, health professionals, and representatives from ID charities) to develop a technology probe of an AAC app to support adults with ID when communicating with GPs. This probe will be embedded in future user-centered design sessions involving participants with mild ID to ensure that the representative requirements for the proposed app are established. Consequently, the lessons disseminated by Gibson et al [37-39] are likely to be premature and subject to change based on the views of the target stakeholders.

##### Participants Involved in the Evaluation of an Intervention

In contrast, participants with mild ID contributed highly to most studies focusing on the evaluation of an intervention [27-29,31-33,35,41]. The only exceptions were the evaluation of a health passport by Heifetz and Lunsky [36], in which only 3 participants with ID completed the feedback questionnaire compared with 25 family members or support workers, and the evaluation of the Comprehensive Health Assessment Program (CHAP) by Lennox et al [29], where the views of practitioners were sought exclusively. A study (Turk et al [33]) reported that a high number of participants with ID (35⁄108, 32.4%) dropped out before completion. This was attributed to people with ID being more likely to refuse follow-up interviews as well as having a higher probability of changing GPs than the general population, meaning they were exempt from the study.

Although people with ID were prevalent throughout the evaluations, only 4 of the articles offered concrete or partial statistics on the etiology of their participants’ disability [27,29,32,33]. As such, we were unable to decipher the characteristics of 72.5% (384/530) of the participants with ID involved in the evaluation studies. In total, 18.5% (98/530) had Down Syndrome [27-29,32,33]; 3.9% (21/530) had autism [33], 3% (16/530) had cerebral palsy [33]; and 2.1% (11/530) had other congenital factors, perinatal birth problems, or epilepsy [33]. The authors noted that cerebral palsy and epilepsy are not often a direct cause of ID but instead coincide with this condition. Nevertheless, we have included them to provide an accurate summary of the participant characteristics reported by the identified studies. Lennox et al [32] primarily measured the severity, but not the cause, of ID present in their participants and found that 44.2% (107/242) had mild or moderate ID and 26.5% (62/242) had severe ID, whereas 30.2% (73/242) participants had an unknown level of ID. Jones and Kerr [27] also followed the same approach, with 25.2% (28/111) of their participants having mild or moderate ID and 35.1% (39/111) having severe ID. Consequently, researchers must provide a consistent, in-depth description of the populations targeted by their studies to increase the generalizability of their findings.

### Thematic Analysis

#### Communication Barriers and Facilitators

Several studies performed qualitative investigations on the barriers and facilitators to effective communication between health professionals and patients with mild ID. Their findings primarily align with the literature (such as the studies by Alborz et al [1], Krahn et al [2], Ali et al [3], Murphy [13], Hemsley and Balandin [43], Selick [49], Chew et al [50], and Pelleboer-Gunnink [51]) and highlight the factors being targeted by the aids introduced in theme 2—*Technological Aids*.

#### Organizational Barriers and Facilitators

##### Collation of Data

Both Raemy and Paignon [40] and Jones and Kerr [27] suggested that a limited collation of health care data regarding ID was a major barrier for patients’ access to effective services. Raemy and Paignon [40] revealed that Switzerland is yet to implement a national policy regarding the health needs of people with intellectual or developmental disabilities, meaning that institutions are not expected to record the details of a patient’s ID, nor have appropriate strategies in place to do so. As such, medical professionals may remain unaware of their patients’ additional needs and therefore fail to conduct the recommended reasonable adjustments to their consultation methods. In addition, the recruitment pathways available to researchers are impacted considerably, as highlighted by Raemy and Paignon [40], who were forced to identify participants via residential accommodation.

Jones and Kerr [27] also acknowledged that it might be difficult for institutions to recognize patients with milder ID. They expected to locate approximately 150 registered patients with ID across 5 GP practices (based on national figures) throughout their study, yet could only identify 39. Consequently, there may be a hidden population of patients with mild ID who are unable to receive the same benefits as those known to medical professionals.

##### Collaboration

In addition to the lack of guidance from national strategies, local health care infrastructure may impede collaboration between medical professionals treating patients with ID. Fragmentation of care was recognized by Bell [35] and Heifetz and Lunsky [36], stemming from a lack of coordination across faculties [35,36] and between health care organizations and social care [36]. As such, people with ID are less likely to receive optimal care because they are prone to developing comorbidities [52], which require treatment from a variety of specialists. Furthermore, patients might find it difficult to adapt to the procedures employed by separate institutions if they are not standardized.

In addition, Heifetz and Lunsky [36] noted that there might be some resistance to agencies moving away from their own practices and instead adopting standard processes or tools, even if there are clear benefits of doing so. In such cases, it is important to establish a champion who can provide strong leadership in overseeing the adoption of the intervention, which may include scheduling regular feedback meetings with stakeholders and periodically reviewing the positive effects of the intervention. This is particularly important in projects where benefits are not immediately clear [36].

##### Time

Dodd and Brunker [28] and Ramey and Paignon [40] highlighted the impact time constraints might have on consultations involving patients with ID. First, Dodd and Brunker [28] suggested that this population is often rushed to communicate their health needs to practitioners, which opens up the possibility of caregivers becoming overinvolved to ensure everything is addressed. As such, the accuracy of the information being provided may be significantly reduced (see the *Support* section). Instead, caregivers should aim to remain in a purely supportive role and encourage patients to proceed at their own pace while interacting with a doctor [28]. In addition, Raemy and Paignon [40] observed that time constraints, particularly in emergency situations, prevented medical professionals from thoroughly exploring all possibilities of an individual’s condition. This included examining the patients often extensive medical histories to gauge whether they had displayed similar symptoms in the past.

#### Education

As discussed previously, medical professionals tend not to be well educated on the health and communication needs of people with ID [11,12]; 4 of the identified studies discussed how this can have a negative impact on the quality of care provided [35,37,38,40]. First, Raemy and Paignon [40] suggested that a lack of knowledge regarding the health trends experienced by people with ID may result in the overshadowing of conditions (ie, the association of a symptom with the disability itself, as opposed to some other disorder) and poor coordination of care. Gibson et al [37,38] and Bell [35] also indicated that insufficient training could affect health professionals’ ability to perform reasonable adjustments, particularly when exchanging information via verbal communication is not an option. Practitioners also complained that they were ill-equipped to overcome the challenging behaviors presented by patients with more severe ID [35].

Due to the shortcomings of undergraduate medical courses [11,12], Bell [35] and Raemy and Paignon [40] called for the introduction of compulsory training sessions on how to treat patients with ID effectively. Bell suggested that this content should focus on the specific communication strategies employed by the ID population, including basic signing systems and other modalities such as imagery [35]. Raemy and Paignon [40] developed a variety of educational resources in conjunction with people with ID to suit the specific needs and workloads of a variety of health professionals. These resources (which ranged from a 15-min educational session to a 5-day training program) covered important aspects such as behavioral traits, including how patients with ID express pain, common health conditions affecting the ID population, and communication strategies to ensure patients are involved in the decisions regarding their care. There is also scope to explore whether training support workers and family members would also have an impact on the health of people with ID [40].

#### Support

There was some disagreement on the impact that external support may have on consultations involving adults with ID. Turk et al [33], Heifetz and Lunsky [36], Gibson et al [37,38], and Lennox et al [30] recognized the important role that caregivers play in empowering individuals with ID to provide their own views. This typically involves serving as a mediator between the patient and health professional to ensure that both sets of stakeholders communicate in a manner understood by the other. In addition, they may be familiar with the patient’s everyday needs and routines [30], which can assist in determining the optimal course of treatment for individuals with ID.

However, the described benefits are largely dependent on the level of involvement a support worker has in the patient’s life. For example, Gibson et al [38], Turk et al [33], and Heifetz and Lunsky [36] noted that some people with ID have to cope with everchanging support workers. Therefore, new staff may be unaware of the person’s health history and specific communication needs, meaning they will have less of an impact on the consultation. Furthermore, there is a possibility that caregivers become overinvolved in the consultation and begin communicating on behalf of the patient [28]. This could reduce the accuracy of the information conveyed because of their own opinions, differing from that of the individual with ID. Finally, Raemy and Paignon [40] demonstrated the advantages of employing more specialized medical professionals to support frontline staff. For 3 years, an ID nurse provided training to less-educated professionals, which improved the standard of care provided to 1017 patients with ID.

#### Person-Centered Care

Lennox et al [30] and Bell [35] noted that optimal care was administered by practitioners who went out of their way to meet individual patient needs. This included simple adjustments such as allowing extra time for the individual to get across their views, being kind and empathetic toward a patient’s situation, interacting directly with a patient rather than their caregiver, using appropriate communication strategies to ensure the patient understands the information conveyed, and looking past a person’s disability to treat them like a human being.

Two strategies were discussed that may assist practitioners in carrying out such adjustments. First, medical professionals should be given access to the personal characteristics of their patients, for example, their preferred method of communicating the terms *yes* and *no*. Second, patients should be encouraged to seek appointments with the same medical professional, thus allowing a relationship to form over time [30,35]. Consequently, practitioners can become increasingly aware of the specific needs of individuals with ID, yet Chinn [41] suggests that this may be difficult for traditional medical professionals in comparison with ID nurses.

#### Technological Aids

In this section, we analyze the various technologies used in the identified studies. To do so, we grouped these technologies into two main categories: low-tech communication aids and high-tech communication aids. We define a low-tech aid as a nonelectronic tool, external to an individual’s body, that assists the user in communicating a message to a relevant partner. In contrast, a high-tech aid is a complex electronic device that permits the storage and retrieval of messages, many of which are used during the formulation of speech output [53].

##### Low-Tech Aids

###### Patient Passports

The bulk of the studies (Brodrick et al [34], Bell [35], Heifetz and Lunsky [36] and Raemy and Paignon [40]) centering on low-tech communication aids used some sort of patient passport. Patient passports encapsulate an individual’s characteristics to assist medical professionals in adjusting their consultation methods to provide consistent, person-centered care. They are typically short in length to allow relevant information to be accessed quickly and may be maintained by all sets of stakeholders involved in a medical consultation, that is, clinicians, support workers, family members, and the patient themselves. As such, they are likely to contain a range of perspectives on the optimal way to interact with a patient with ID, thus increasing the probability of doing so effectively.

The passports implemented shared common features but were often tailored to meet the requirements and infrastructures of the organizations they were employed in. This was demonstrated concretely by Heifetz and Lunsky [36], who developed passports for 3 institutions within the same catchment area. Each institution requested a tool that differed in size (wallet size vs 1 full-page, double-sided tool vs 4 pages) and visual appearance (plain written information vs pictures to complement text). However, all formats summarized information on the same aspects, including the patient’s medical history, baseline behaviors (eg, potential triggers, communication methods, or contingency plans for when the patient becomes agitated), and the emergency contact details of support workers and family members.

Brodrick et al [34] and Bell [35] encapsulated similar details in their double-sided and 3-page patient passports, respectively. Nevertheless, they used color to demonstrate the most relevant aspects required in a critical situation. For example, the medical needs of the patient (eg, existing conditions and allergies) were prioritized by both sets of authors, meaning this information was coded in red to signify its importance. Further information, such as the patient’s environment or support needs—those deemed to be relevant but not critical to the patient’s care—was coded in more neutral colors such as amber and green.

Raemy and Paignon [40] recognized that passports could only be effective if they accompany patients throughout the health care system, a process that may be difficult to achieve using physical resources. Consequently, they developed a digital version and integrated it within their electronic patient data system to increase the portability of the aid produced. Multiple health care professionals may also have access to passports at the same time if required.

###### CHAP or Notes-Based Prompts

Lennox et al [29,32] and Jones and Kerr [27] explored using note-based prompts to support medical professionals in investigating specific areas of a patient’s health. The CHAP [29,32] is composed of a list of screening opportunities and preventive activities commonly used by people with ID. Practitioners then use this information to determine whether appropriate health checks have been carried out periodically with the patient. As a result, the CHAP is less likely to positively affect time-critical environments, such as primary care consultations, where the emphasis is placed on treating the most immediate symptoms present [32]. Instead, it is more suited to interventions such as the ID annual health check, as medical professionals have an extended amount of time to consider all aspects of a patient’s well-being.

In addition to the CHAP, Lennox et al [29] supplied health professionals with a short summary of the recent health trends of people with ID, a strategy they found most convenient to use in general practice. Jones and Kerr [27] also employed a similar approach to encourage practitioners to be vigilant for, and follow-up on, conditions that may otherwise have been missed or overshadowed. They combined such evidence with a synopsis on the best practices to implement when interacting with a patient with ID, thus potentially increasing the amount and accuracy of information being extracted. Nevertheless, they found that the paper-based nature of the aid meant it was not used prominently by health professionals [27] and could therefore be replaced by more appropriate digital solutions.

###### Health Diaries

Lennox et al [30,32] and Turk et al [33] described the development of health care diaries to empower patients with ID to understand their needs better as they progress over time. Once again, all stakeholders were responsible for maintaining the document, meaning that observations on the patient’s well-being could be recorded by health professionals, support workers, family members, or the individual with ID. The approach of Turk et al [33] separated the diary into sections based on the common conditions experienced by people with ID, ranging from everyday ailments to more complex disorders such as epilepsy. There was also space dedicated to the treatments being received by the individual as well as advice on how to live a healthy lifestyle.

The diary of Lennox et al [30,32] was significantly more substantial in that it contained segments on how to improve communication during the consultation, in addition to those focusing on recording health information. These segments were aimed at both the health professional and the individual with ID and included a patient passport, general strategies that practitioners may use to improve the quality of care being provided, and tips for the patient on how to prepare for a consultation, along with several resources to support them during this process, such as picture symbols and pain recording tools. Consequently, the health professional’s knowledge of the patient’s communication or treatment preferences and specific health needs should be notably increased.

###### Easy Read

Dodd and Brunker [28] and Chinn [41] used easy-read documents to support patients with ID in understanding medical conditions or symptoms. *Easy read* is the term given to information resources that have been specifically adapted to suit the complex needs of people with ID. This is primarily achieved through the implementation of short, jargon-free sentences supplemented with identifiable imagery.

In the study of Dodd and Brunker [28], flashcards of various body parts, types and intensities of pain, and periods of time were issued to patients with ID to increase the accuracy of the symptoms being described. The approach by Chinn [41] was different in that she directed medical professionals toward existing easy-read resources on clinical conditions and monitored whether these resources had a direct impact on communication throughout a consultation. The documents included an accessible summary of the effects and potential treatments of a condition. Consequently, they were used as a form of support during situations where a patient could not understand what the practitioner was conveying or was opposed to the course of treatment being offered. Despite the documents being publicly available before the commencement of the study, many of the GPs were largely unfamiliar with such resources, thus potentially limiting their impact on consultations. This contrasted with the more specialized health care professionals (ID nurses) who regularly used, and were involved in the development of, easy-read resources [41].

###### Talking Mats

Bell and Cameron [31] identified Talking Mats as a potential tool for supporting a patient with mild ID in discerning aspects of their mental health—a process that they were finding difficult to overcome using traditional consultation methods. Talking Mats is a communication aid that primarily relies on images to form a concrete representation of an individual’s views. A visual scale was first placed at the top of a physical mat. The discussion was then broken down into manageable topics, and for each topic, the individual should place an image that encapsulates their opinion under the appropriate section of the visual scale. Consequently, the aid is particularly effective for individuals who lack the social skills to converse with authoritative figures, as it lifts the burden of direct interactions [31]. In addition, Talking Mats may provide a voice for those who are unable to communicate verbally, thus increasing their participation in decisions regarding their care.

##### High-Tech Aids

Only 1 set of authors (Gibson et al [37-39]) explored the development of high-tech aids to support patients with mild ID when communicating with medical professionals. They proposed a digital questionnaire based on the most common medical conditions experienced by people with ID. Each question should be presented using the easy-read format discussed above to increase the probability of users selecting the symptoms they are experiencing. In addition, any information extracted from the patient should be used to influence the future questions presented, thus ensuring that the questionnaire is tailored to their own health care needs. The app should also be customizable to account for the patient’s accessibility profile and may be combined with other AAC strategies, such as patient passports, to increase the quality of care being provided [39].

Extracting symptoms from patients with ID before the consultation may have multiple advantages. The results may be used as a referent by the patient when presenting their views to health professionals; time constraints may be reduced with the practitioner able to build upon preselected information; and finally, there may be increased exposure to commonly overshadowed conditions [37-39]. However, without a concrete evaluation (which includes the involvement of target stakeholders), such benefits may be speculative, with the lessons disseminated by Gibson et al likely to change as further studies are carried out.

#### Communication Modalities

In total, 67% (10/15) of studies, including the studies by Dodd and Brunker [28], Lennox et al [29,30,32], Bell and Cameron [31], Heifetz and Lunsky [36], Gibson et al [37-39], and Chinn [41], described their implemented technologies well enough for the authors to determine the range of communication modalities employed.

##### Imagery

The bulk of the articles discussed the importance of imagery in supporting patients with ID to understand and communicate about their symptoms. Nevertheless, the depth and context of the use of medical images differed. For example, Bell and Cameron’s [31] application of Talking Mats resulted in a patient with mild ID providing information on their psychological health via the development of a pictorial framework. This, therefore broke the reliance on disseminating information through speech, with the individual only being required to elaborate on those selections that were unclear or of particular importance to their diagnosis. The visual feedback offered by the mat also enabled the patient to reflect on and refine their selections, thus increasing the quality and quantity of information provided.

Lennox et al [30,32] and Dodd and Brunker’s [28] use of imagery was less extensive in that their resources enhanced an individual’s communicative abilities instead of primarily replacing them. In both cases, this involved developing colorful pictures to support a patient with ID in expressing pain symptoms, including its site, severity, [28,30,32], intensity, and duration [28]. Heifetz and Lunsky [36] also found it beneficial to include a photograph of the patient in any resources used, to give practitioners a reference of how they should look while healthy.

Finally, the imagery employment of Chinn [41] and Gibson et al [37-39] was aimed at enhancing patients’ understanding of relevant medical information. In the study by Chinn [41], health professionals used easy-read documents at times when a patient was unable to understand what was being conveyed or disagreed with the course of treatment proposed. These documents contained information on the manifestation, effects, and possible treatments of a condition and were made more accessible to the ID population by introducing imagery. Therefore, the ability of patients to be involved in decisions regarding their care should have increased. Gibson et al [37-39] applied a similar strategy during the design of a clinical AAC tablet app, with images being used to supplement the patients’ understanding of the symptoms presented as part of a medical questionnaire. In addition, symbols were used to indicate the functionality of the buttons embedded in the app’s user interface, albeit varying degrees of success [37,38].

Despite their reliance on imagery throughout the technologies implemented, none of the authors discussed the design decisions taken during the development of such resources. Furthermore, none of the image sets were made publicly available, which impacts the ability of researchers to reuse them or indeed create their own. Lennox et al [30] also noted that images could be expensive and time-consuming to produce, and this could be a problem considering that a one-size-fits-all approach is unlikely to be effective for the ID population [37-39]. For example, some patients may already use Makaton symbols [54] in their everyday lives, and therefore expect a similar style of image to be employed, whereas others might find realistic photographs to be more relatable.

##### Text and Speech

In total, 5 studies (Lennox et al [30], Gibson et al [37-39], and Chinn [41]) indicated that written information, enhanced by identifiable imagery, provided patients with an accessible means of two-way communication. Gibson et al [37-39] went one step further and suggested that the playback of textual information should also be incorporated, where possible, to ensure illiterate or semiliterate users are not disadvantaged in any way. Therefore, targeting a range of modalities ensures that information is presented in a variety of different manners, with the individual able to use the form that makes the most sense to them in each scenario. For example, a patient with ID may prefer to use images when receiving information but also has the option to fall back on the text when a particular image is unclear.

While developing textual information, Chinn [41] and Gibson et al [37-39] emphasized the importance of following accessible language guidelines, such as National Health Service England’s [55]. This included the use of plain and simple sentences that focused on solitary ideas. However, Gibson et al [38] also recognized that some complex terminology, such as medication brand names, was crucial to patient comprehension, meaning it is important to develop such resources in conjunction with target stakeholders to ensure their needs are met.

When presenting questions to patients with mild ID, different strategies were employed depending on the context of the consultation and the technologies used. For example, Bell and Cameron [31] primarily presented open-ended questions when using Talking Mats to establish the factors having a negative impact on the psychological health of a patient with ID. They felt that open-ended questions could improve the quality and depth of information being extracted, although they recognized that the ID population might have greater difficulty in constructing responses to them. In contrast, Gibson et al [37-39] used closed questions that focused on a narrow range of medical symptoms, thus enabling them to break the consultation process down into manageable steps while building an overall picture of the patient’s health care needs.

##### Training

Bell also suggested that health care professionals remain undereducated on the communication strategies employed by patients with ID [35]. Consequently, she called for the enhancement of existing training programs to include information on how to effectively target a range of communication modalities instead of just using speech. This included basic signing systems such as Makaton [54,56], in addition to simplified language and imagery.

#### Evaluation and Impact of the Technologies

In this section, we analyze the evaluation techniques employed in the identified studies. The perceived impact of the technologies that emerged as a result of these evaluations will also be discussed.

##### Qualitative Evaluations

Most studies primarily used qualitative methods to evaluate the effect of their technologies on current practice; this included interviews, focus groups, and questionnaires [28-31,34-36,40], the analysis of a reflective journal [35], posttask walkthroughs [37,38], and conversational analysis of the interactions between health professionals and patients with ID [41].

###### Interviews, Focus Groups, and Questionnaires

####### The CHAP

Lennox et al [29] initially assessed the benefits of their CHAP, which included a checklist of preventive activities, a synopsis of the literature on the current health trends of the ID population, and a health record audit tool, by issuing a self-evaluation form to the practitioners involved in the study. Of the 45 GPs who agreed to participate, only 15 (33.33%) completed all the study components. This, combined with the lack of involvement of the 38 patients with ID in the intervention evaluation phase, significantly restricts the strengths of the conclusions made, as highlighted by the fact that only descriptive results were reported. In terms of effectiveness, the GPs reported that all interventions were beneficial in assisting their provision of care. Nevertheless, the synopsis of the literature was most productive in improving their knowledge of the health demographics of people with ID and was considered the most practical to use [29]. The checklist was most likely to raise awareness of the health needs of the patient and therefore prompted the greatest amount of action that may not have been carried out otherwise. Communication was reported to have increased between carers, hospitals, and specialists, as were consultation times, although no quantitative measures were carried out to confirm this.

####### Ask it Health Diary

Lennox et al [30] employed a similar evaluation form to determine the appropriateness of an educational session that preceded the implementation of a health advocacy diary. The finer details of the form were not disclosed, yet the feedback indicated that the session was useful in reinforcing the responsibilities of both the patient and the health professional. In addition, the session also introduced the steps involved in becoming an effective advocate. A short pilot study was conducted with the following 2 groups to evaluate the health diary: (1) 19 parents of adults with ID who used a nongovernmental support service and (2) 7 people with ID who used a nongovernmental accommodation service. The participants took part in the educational sessions mentioned above and were then issued with the health diary. Next, they were required to familiarize themselves with the tool for 2 weeks before completing an interview on the phone or in person, the protocol of which was not described. The qualitative data indicated that the diary improved the advocacy skills of two-thirds of the participants and improved their relationship with the GP in 50% of cases. The results were also used to improve the technology before a more thorough evaluation was conducted in [32].

####### Talking Mats

Bell and Cameron [31] conducted 2 separate interviews to validate the health information extracted from a patient with mild ID using Talking Mats. The patient’s concerns extracted during these interviews were collated into a single document, with arrows being included to show how they had changed. This information was then passed on to the individual’s support worker to ensure that actionable change was carried out to improve their mental health. Bell and Cameron [31] found that the Talking Mats framework made it possible to “extend the use of therapies that rely heavily on verbal communication to those people who not only ﬁnd verbal communication difﬁcult in a general sense but also in a speciﬁc situational sense.” Visual feedback, along with the open-ended questions presented, may also increase the depth and quality of the information being extracted.

####### Easy-Read Communication Cards

Dodd and Brunker [28] issued a questionnaire at the start of their project to determine the health advocacy skills of 10 patients with ID. After 6 months of using easy-read communication cards and participating in the accompanying training sessions, participants were required to redo the questionnaire to determine if their skills had improved. Brief multiple-choice questionnaires were also completed by the participants, GPs, and key workers each time a participant became ill or was in pain and visited their doctor. In total, 3 follow-up evaluation cards were completed by the participants involved, meaning that the authors were only able to provide tenuous remarks regarding the feedback received [28]. The benefits reported included an increase in knowledge on recognizing the signs of being unwell and what to do when ill, an increase in two-way communication using the pictorial aids issued, and an increase in the ability of the patients to be involved in the decisions regarding their care. Nevertheless, there was some variance in the results extracted, with only those participants who used the aid regularly with their support worker or doctor demonstrating increased retention of health care information.

####### Patient Passports

Heifetz and Lunsky [36] also used both questionnaires and interviews to evaluate patient passports across 3 institutions in Canada. Their descriptions of the protocols employed were more complete, thus increasing the replicability of their findings. A total of 18 semistructured interviews were conducted on the phone with a variety of stakeholders, including hospital clinical staff, community health and ID service providers, community-based health care coordinators, and 1 parent. Participants with ID were not included in this stage, as the focus of the interviews was on the implementation of the passports rather than their use. Instead, the ID population’s views were extracted using a questionnaire, along with support workers and family members, to determine the fit and user-friendliness of the passport and its potential benefits. Both closed- and open-ended questions were used to achieve this.

Overall, 75% (21/28) of the participants involved in the questionnaire felt that the tool provided health care professionals with relevant background information on the patient. In total, 65% (18/28) suggested that such an approach can assist practitioners in carrying out reasonable adjustments to their consultation methods, with 79% (18/28) recognizing an improvement in communication between all stakeholders involved in a consultation. Consequently, the tool has the potential to support practitioners in conducting better-informed health care decisions. Nevertheless, these results may be speculative as only 3 of the participants who completed the questionnaire had ID, 25 did not have ID, and 82% (23/28) had no experience in using the aid within a health care context. The interviews also highlighted the variable degree to which passports were adopted across each institution. Strong leadership in monitoring and educating professionals on using tools has been reported as increasing community awareness and buy-in [36].

Brodrick et al [34] conducted a short pilot study of a 1-page patient passport across 2 sites in England in October 2009. Residential managers from each service were trained using passports before introducing the aid to frontline care staff. During 1 month, 150 passports were produced, with both the researchers and residential managers remaining on hand to provide additional training and support. Quality checks were carried out on these resources, and a final round of focus groups was conducted at the end of the pilot phase to obtain feedback from the health care staff. Nonetheless, the components being reviewed throughout the quality checks and the tasks employed in the focus groups were not reported. The potential benefits of the passport were similar to those reported by Heifetz and Lunsky [36] in that it provided staff with the necessary information to deliver person-centered care. Passports also increased the continuity of care as patients moved across departments while promoting collaboration between health care providers. However, their initial quality was extremely variable and only improved once extra training and support were provided, along with passports deemed to be of high caliber.

###### Reflexive Journal Analysis

Bell [35] used multiple methods to evaluate their version of a patient’s passport. A variety of perspectives were extracted, thus improving the strengths of the findings obtained via data triangulation. First, 12 family caregivers and health and social care staff participated in a series of semistructured interviews to determine their experiences using the passport. In addition, a focus group involving 8 adults with ID was conducted, with emphasis being placed on aspects that had, or had not, helped them feel comfortable in a hospital context. Nevertheless, only 1 participant had experience using the passport employed, which potentially limits the impact of the findings from this part of the study. Finally, Bell [35] observed and recorded notes on passports being implemented in practice, which was analyzed using a reflexive process. As with Heifetz and Lunsky [36] and Brodrick et al [34], increased collaboration and continuity of care were recognized across multiple health care providers.

###### Conversational Analysis

Chinn [41] recorded the interactions between health professionals and patients with ID to determine the effects easy-read information sheets had on consultations. A total of 41 recordings were made, 32 of which involved a patient with ID attending a health check with primary care clinicians and 9 with specialist ID nurses. Conversational analysis was then used to examine the interactional micropractices that framed literacy events involving easy-read resources. Reflective interviews were also conducted with a subset of the participants (9 patients and 9 health professionals) to determine the reasons behind certain actions. The study by Chinn [41] was carried out in the context of annual health checks to ensure the identification of appropriate participants. However, this environment restricted the opportunity for health professionals to introduce easy-read information sheets, as highlighted by their visibility in just 22% (7/32) of the appointments recorded. The ID nurses involved were also far more likely to use the information sheets than the GPs (because of their specialized skills) despite Chinn’s best effort to educate the participants on the benefits of such resources. When used, the easy-read information sheets effectively supported the medical professional to offer unsolicited advice, particularly when patients were resistant to change. This was because of the aid reinforcing the practitioner’s views and reminding them of important aspects to forward on to the patient.

###### Posttask Walkthrough

Gibson et al conducted posttask walkthroughs with 4 experts in ID to ensure that the technology probe of a clinical AAC tablet was accessible to the target population [37,38]. The experts were required to select various symptoms within the probe before answering questions on their experience with the app. Particular attention was paid to any area of interest noted by the researchers during the experts’ interactions. The benefits of the app listed by the participants included an increase in communication via the use of an accessible list of symptoms as a referent, a rise in awareness of the conditions commonly overshadowed by practitioners, and the mitigation of time constraints by providing information to the GP before the consultation. Nonetheless, such benefits may be premature, with Gibson et al revealing their intentions to extract the views of health professionals and adults with mild ID during future work before carrying out a pilot study within the clinical environment [37,38].

##### Quantitative Evaluations

Only 3 studies [27,32,33] used quantitative methods, via randomized controlled trials (RCTs), to determine the effect of their interventions on current practice.

###### Ask It Health Diary and CHAP

Lennox et al [32] followed on from their earlier studies [29,30] to perform a clustered RCT with people with ID living in private dwellings throughout the Greater Brisbane area of Australia. They examined the effect of their interventions using a 2×2 factorial design, with the units of randomization being assigned to clusters of participants interlinked by sharing a GP practice. These clusters were organized into 4 blocks according to their size; 1 cluster from each block was then assigned to a factorial group by a statistician using computer-generated random numbers. The effects of the interventions on clinical activity (eg, health promotion and disease prevention) were measured for 12 months and compared with the same activities in the preceding year.

The CHAP had a statistically significant effect on health promotion, disease prevention, and case finding activities across a number of components. Outcomes related to sensory systems (eg, hearing and vision tests) increased, as did all 5 of the immunizations highlighted by the program. There was also a substantial increase in the number of patients who underwent weight measurements. There were no significant changes in the measured outcomes of the group assigned to the *Ask It* health diary alone, with only modest effects being noted on epilepsy review and constipation investigation. This contrasts with the findings of [30], which suggested that the health diary could lead to an improvement in the patient’s health advocacy skills, and as such, increase the number of conditions being identified. Lennox et al suggested that the trial may have been too short to recognize the true benefits of the diary [32].

###### Notes-Based Prompt

Jones and Kerr [27] also used an RCT to evaluate their note-based prompt, a tool that was similar to the CHAP program described above. A total of 5 primary care practices participated in the study and identified 88 patients with ID who were randomly allocated to the active or control group. The active group had access to the prompt immediately, whereas the control group endured an embargo for 6 months. After the initial 6-month period, data were collected on a wide range of variables related to health promotion, consultation patterns, and physical, psychological, and social well-being. This was compared with information on consultation patterns during the previous 4 years as well as life-long records of general health issues. In contrast to Lennox et al [32], no significant differences were observed in consultation patterns (location, nature, and outcome) or health promotion. Jones and Kerr [27] attributed this to the paper-based nature of the aid, with medical professionals preferring to use digital resources. In addition, they suggested that without statutory regulations and considering the current workloads experienced by GPs, screening opportunities are unlikely to be carried out on an opportunistic basis.

###### Hand-Held Health Record or Diary

Finally, Turk et al employed an RCT to evaluate their hand-held health diary [33]. A total of 40 primary care practices were randomized to the control or implementation groups, with 163 patients with ID completing all stages of the trial. Initial interviews were carried out with patients and caregivers to determine aspects such as basic background information, knowledge of health problems and medical terminology, information on GP visits in the past year, and whether specific health checks were up to date. Follow-up interviews were then conducted 1 year after the study’s start date and were identical to the initial interviews, except that additional questions were asked about the individuals’ experience with the health diary where appropriate. Upon completion of the study, a nurse researcher accessed the patients’ medical records from a year before the initial interviews up to the time of the follow-up interview to measure a number of health-related outcomes.

Similar to Lennox et al [32], no statistically significant outcomes were achieved by the hand-held health diary [33]. However, there were some improvements concerning the number of GP visits per year (an increase of 1.4), the ability of patients to report health-related problems, and their ability to recognize medical jargon. The qualitative data extracted during the follow-up interviews indicated that only 18% (10/56) of the patients with ID involved in the intervention group used the diary, and 39% (22/56) of caregivers used it on behalf of the patient. This may partially explain the limited impact that the diary had on consultation patterns, the impact that was attributed to a high turnover in support staff, and other factors such as carers forgetting it, being too busy, or being concerned about taking up the GPs’ time. Nevertheless, those who had used the diary generally expressed satisfaction with it and suggested that it helped them know more about the patient’s health and was useful during visits to the GP or hospital.

Raemy and Paignon’s [40] evaluation phase is currently in process; therefore, no concrete results have been reported. In addition, the study by Gibson et al [39] only focused on the extraction of design requirements, meaning no evaluation was conducted.

## Discussion

### Principal Findings

Despite communication barriers being well recognized within the literature (eg, in the studies by Alborz et al [1], Krahn et al [2], Ali et al [3], Hanlon et al [5], and Hemsley and Balandin [43]), little is known about the use of technology to support the exchange of information between patients with mild ID and medical professionals. Our review therefore maps the literature within this domain while exposing potential gaps that may be addressed in future work. We identified only 15 studies focusing on the development and/or implementation of AAC devices, with most investigating one-way communication aids [27,29,32-36,40]. Notes-based prompts (Jones and Kerr [27], Lennox et al [29,32]) were statistically significant in increasing the number of targeted checks performed by medical professionals in problematic areas, such as hearing difficulties [32]. Passports and health diaries (Turk et al [33], Brodrick et al [34], Bell [35], Heifetz and Lunksy [36], and Raemy and Paignon [40]) aimed to increase practitioners’ knowledge of their patients’ medical and communication needs, thereby facilitating reasonable adjustments and recognizing commonly overshadowed conditions. However, these interventions centered on the way medical professionals present information to their patients instead of empowering individuals with mild ID to take an active role in their care. This goes against Chinn’s [22] view that the best outcomes for consultations occur when both parties receive support to enhance communication.

In contrast, the interventions described by Dodd and Brunker [28], Lennox et al [30], Gibson et al [37-39], and Chinn [41] aimed to facilitate improved two-way communication. Images of symptoms and body parts were used in multiple ways by Dodd and Brunker [28], Lennox et al [30], and Bell and Cameron [35] to promote discussion on such topics. Easy-read resources were also embedded in consultations to enhance patients with mild ID knowledge of certain conditions or procedures, thus improving their ability to provide informed consent [41]. Finally, Gibson et al [37-39] investigated the use of digital questionnaires to produce an easy-read summary of the main symptoms experienced by an individual with ID. Both the patient and the medical professional may then build upon this summary throughout the consultation. Ensuring that all stakeholders share a mutual understanding of the clinical information being discussed is likely to lead to more accurate diagnoses being carried out. As such, the authors agree with Chinn [22] that greater emphasis should be placed on developing and evaluating two-way communication aids.

Nonetheless, one-way communication aids, particularly patient passports, still play a role in environments that are time-critical (eg, accident and emergency) or difficult to navigate (eg, large-scale hospitals, multiple wards) to ensure consistent care is administered [34,35]. However, Hemsley and Balandin [43] recognized that overly long summaries of an individual’s needs might result in medical professionals ignoring such information, with the patient having to repeat themselves on multiple occasions. This could, therefore, explain the change in focus toward 1-page patient passports [34-36].

### Systemic Change

The bulk of the communication barriers discussed within our review match the findings of Hemsley and Balandin [43]. However, not all may be alleviated by the simple introduction of AAC technologies and require much more systemic changes. Hemsley and Balandin [43] noted that government and health care agencies must do more to reduce the inequalities experienced by patients with complex communication needs. An instance in which this is abundantly clear is Switzerland’s failure to implement a national ID strategy, meaning that institutions lack the appropriate guidance and resources to treat patients with ID effectively [36]. Therefore, additional services, systems, and policies [43] must be developed on a national scale to encourage improved person-centered care. Hemsley and Balandin [43] highlighted various aspects that must be considered during this process: (1) increasing the knowledge of health care staff on effective communication strategies, (2) extending the time available to consult with patients with complex communication needs, (3) increasing interagency collaboration to ensure patients are able to take the optimal pathway through complex health systems, (4) clearly defining the role of caregivers, and (5) increasing access to and encouraging the use of AAC devices within consultations. The studies identified in our review also suggested that targeted health checks [27,29] and the employment of specialized professionals to support frontline staff, such as ID nurses, could have serious benefits for the well-being of the ID population. Introducing statutory regulations should also help ensure that interventions are used within the practice—a problem identified by some of the reviewed studies [27,35].

Finally, the health inequalities experienced by patients with milder ID may be exacerbated because of the *hidden* nature of their disability [27]. Their symptoms were not as prominent as those of moderate or severe ID, indicating that their diagnosis could be delayed or missed entirely. As such, medical professionals may continue to employ inappropriate consultation techniques because of their ignorance of their patients’ additional needs. Consequently, practices should employ ID registers [57] to ensure that medical professionals are aware of their need to conduct reasonable adjustments. In addition, greater emphasis must be placed on strategies to identify people with mild ID.

### Study Limitations and Recommendations for Future Work

Our review is the first to explore the types of AAC technologies available to patients with mild ID during clinical consultations. Despite the abundance of evidence detailing the health inequalities experienced by patients with ID, we highlight the limited extent of research being carried out in this area. Further investigations into the potential of two-way communication aids in increasing the health advocacy skills of this population must be conducted to emphasize the use of high-tech aids, as they can be adapted to the working routines of medical professionals. Quantitative measures must also be employed to determine clinical advantages. Nevertheless, this study is a scoping review, not a systematic review, and therefore has some limitations. First, the searches were restricted to 3 primary databases, meaning that relevant literature may have been omitted. Second, only articles published in English were considered, which may explain why the identified studies were carried out by members of the OECD. There is also scope to explore the use of AAC devices to improve the health of other populations, such as those with more severe ID [58] or children [59-61].

### Conclusions

Communication aids have the potential to provide immediate health benefits to people with ID in the absence of wholesale changes being carried out in organizational procedures, such as undergraduate training. Therefore, this review summarizes the use of low- and high-tech communication aids by adults with mild ID in the context of primary and secondary care. The advantages of the aids used included assisting medical professionals in making reasonable adjustments to their consultation methods by providing them with personal information on the patient, increasing two-way communication, and enhancing practitioners’ awareness of the health trends experienced by people with ID. Nevertheless, there were some deficiencies in the methods used by the identified studies that limited the impact and generalizability of the conclusions. Areas that require further consideration include using quantitative methods during RCTs to determine the true benefits of the aids in a clinical context and additional investigations regarding high-tech two-way communication aids.

## Appendix

Multimedia Appendix 1 In-depth overview of identified studies.

## Abbreviations

AAC: alternative and augmentative communication
CHAP: Comprehensive Health Assessment Program
GP: general practitioner
ID: intellectual disability
OECD: Organization for Economic Cooperation and Development
PRISMA: Preferred Reporting Items for Systematic Reviews and Meta-Analyses
RCT: randomized controlled trial

## References

[1] Alborz A, McNally R, Glendinning C (2005). Access to health care for people with learning disabilities in the UK: mapping the issues and reviewing the evidence. J Health Serv Res Policy.

[2] Krahn G, Hammond L, Turner A (2006). A cascade of disparities: health and health care access for people with intellectual disabilities. Ment Retard Dev Disabil Res Rev.

[3] Ali A, Scior K, Ratti V, Strydom A, King M, Hassiotis A (2013). Discrimination and other barriers to accessing health care: perspectives of patients with mild and moderate intellectual disability and their carers. PLoS One.

[4] Heslop P, Blair P, Fleming P, Hoghton M, Marriott A, Russ L (2013). Confidential Inquiry into premature deaths of people with learning disabilities (Final report). CIPOLD, University of Bristol, and Department of Health.

[5] Hanlon P, MacDonald S, Wood K, Allan L, Cooper SA (2018). Long-term condition management in adults with intellectual disability in primary care: a systematic review. BJGP Open.

[6] Lauer E, McCallion P (2015). Mortality of people with intellectual and developmental disabilities from select US state disability service systems and medical claims data. J Appl Res Intellect Disabil.

[7] (2019). Health and care of people with learning disabilities: 2017-18. NHS Digital.

[8] (2013). Good practice guidelines for staff who work with people with learning disabilities. The Hillingdon Hospitals - NHS Foundation Trust.

[9] (2021). Learning disabilities - communication with patients. General Medical Council.

[10] (2018). Guidelines on caring for people with a learning disability in general hospital settings. Regulation and Quality Improvement Authority.

[11] Phillips A, Morrison J, Davis RW (2004). General practitioners' educational needs in intellectual disability health. J Intellect Disabil Res.

[12] Trollor J, Ruffell B, Tracy J, Torr JJ, Durvasula S, Iacono T, Eagleson C, Lennox N (2016). Intellectual disability health content within medical curriculum: an audit of what our future doctors are taught. BMC Med Educ.

[13] Murphy J (2006). Perceptions of communication between people with communication disability and general practice staff. Health Expect.

[14] Shanafelt TD, Hasan O, Dyrbye LN, Sinsky C, Satele D, Sloan J, West CP (2015). Changes in burnout and satisfaction with work-life balance in physicians and the general US working population between 2011 and 2014. Mayo Clin Proc.

[15] Hall LH, Johnson J, Watt I, O’Connor DB (2019). Association of GP wellbeing and burnout with patient safety in UK primary care: a cross-sectional survey. Br J Gen Pract.

[16] Jones-Berry S (2016). Not enough learning disability nurses for future patients. Nursing Standard.

[17] Joddrell P, Astell AJ (2016). Studies involving people with dementia and touchscreen technology: a literature review. JMIR Rehabil Assist Technol.

[18] Linskell J, Bouamrane MM (2012). Assisted-living spaces for end-users with complex needs: a proposed implementation and delivery model. Health Informatics J.

[19] den Brok W L J E, Sterkenburg P S (2015). Self-controlled technologies to support skill attainment in persons with an autism spectrum disorder and/or an intellectual disability: a systematic literature review. Disabil Rehabil Assist Technol.

[20] (2021). Definition: intellectual disability. World Health Organization.

[21] Hammill DD (1990). On defining learning disabilities: an emerging consensus. J Learn Disabil.

[22] Chinn D (2017). Review of interventions to enhance the health communication of people with intellectual disabilities: a communicative health literacy perspective. J Appl Res Intellect Disabil.

[23] Arksey H, O'Malley L (2005). Scoping studies: towards a methodological framework. Int J Soc Res Methodol.

[24] Haddaway NR, Collins AM, Coughlin D, Kirk S (2015). The role of Google scholar in evidence reviews and its applicability to grey literature searching. PLoS One.

[25] Liberati A, Altman DG, Tetzlaff J, Mulrow C, Gøtzsche PC, Ioannidis JP, Clarke M, Devereaux PJ, Kleijnen J, Moher D (2009). The PRISMA statement for reporting systematic reviews and meta-analyses of studies that evaluate health care interventions: explanation and elaboration. PLoS Med.

[26] Methley A, Campbell S, Chew-Graham C, McNally R, Cheraghi-Sohi S (2014). PICO, PICOS and SPIDER: a comparison study of specificity and sensitivity in three search tools for qualitative systematic reviews. BMC Health Serv Res.

[27] Jones RG, Kerr MP (1997). A randomized control trial of an opportunistic health screening tool in primary care for people with intellectual disability. J Intellect Disabil Res.

[28] Dodd K, Brunker J (1999). ‘Feeling Poorly’: report of a pilot study aimed to increase the ability of people with learning disabilities to understand and communicate about physical illness. Br J Learn Disabil.

[29] Lennox N, Green M, Diggens J, Ugoni A (2001). Audit and comprehensive health assessment programme in the primary healthcare of adults with intellectual disability: a pilot study. J Intellect Disabil Res.

[30] Lennox N, Taylor M, Rey-Conde T, Bain C, Boyle FM, Purdie DM (2004). ask for it: development of a health advocacy intervention for adults with intellectual disability and their general practitioners. Health Promot Int.

[31] Bell DM, Cameron L (2008). From Dare I say … ? to I dare say: a case example illustrating the extension of the use of Talking Mats to people with learning disabilities who are able to speak well but unwilling to do so. Br J Learning Disab.

[32] Lennox N, Bain C, Rey-Conde T, Taylor M, Boyle FM, Purdie DM, Ware RS (2010). Cluster randomized-controlled trial of interventions to improve health for adults with intellectual disability who live in private dwellings. J Appl Res Intellect Disabil.

[33] Turk V, Burchell S, Burrha S, Corney R, Elliott S, Kerry S, Molloy C, Painter K (2010). An evaluation of the implementation of hand held health records with adults with learning disabilities: a cluster randomized controlled trial. J Appl Res Intellect Disabil.

[34] Brodrick D, Lewis D, Worth A, Marland A (2011). One-page patient passport for people with learning disabilities. Nurs Stand.

[35] Bell Ruth (2012). Does he have sugar in his tea? Communication between people with learning disabilities, their carers and hospital staff. Tizard Learning Disability Rev.

[36] Heifetz M, Lunsky Y (2018). Implementation and evaluation of health passport communication tools in emergency departments. Res Dev Disabil.

[37] Gibson RC, Bouamrane MM, Dunlop MD (2018). Mobile support for adults with mild learning disabilities during clinical consultations. Proceedings of the 20th International Conference on Human-Computer Interaction with Mobile Devices and Services.

[38] Gibson RC, Bouamrane MM, Dunlop MD (2019). Design requirements for a digital aid to support adults with mild learning disabilities during clinical consultations: qualitative study with experts. JMIR Rehabil Assist Technol.

[39] Gibson RC, Bouamrane MM, Dunlop MD (2019). Experts views on the use of mobile devices to support patients with mild learning disabilities during clinical consultations. Stud Health Technol Inform.

[40] Raemy SL, Paignon A (2019). Providing equity of care for patients with intellectual and developmental disabilities in Western Switzerland: a descriptive intervention in a University Hospital. Int J Equity Health.

[41] Chinn D (2020). An empirical examination of the use of Easy Read health information in health consultations involving patients with intellectual disabilities. J Appl Res Intellect Disabil.

[42] Gale NK, Heath G, Cameron E, Rashid S, Redwood S (2013). Using the framework method for the analysis of qualitative data in multi-disciplinary health research. BMC Med Res Methodol.

[43] Hemsley B, Balandin S (2014). A metasynthesis of patient-provider communication in hospital for patients with severe communication disabilities: informing new translational research. Augment Altern Commun.

[44] Baxter S, Enderby P, Evans P, Judge S (2012). Barriers and facilitators to the use of high-technology augmentative and alternative communication devices: a systematic review and qualitative synthesis. Int J Lang Commun Disord.

[45] (2021). Alternative and Augmentative Communication Technologies to Support Adults with Mild Intellectual Disabilities during Clinical Consultations: Scoping Review Charted Data. University of Strathclyde.

[46] (2019). The keys to life - unlocking futures for people with learning disabilities implementation framework and priorities 2019-2021. Scottish Commission for Learning Disability.

[47] (2015). Who Global Disability Action Plan, 2014-2021: Better Health for All People With Disability.

[48] Desmond D, Layton N, Bentley J, Boot FH, Borg J, Dhungana BM, Gallagher P, Gitlow L, Gowran RJ, Groce N, Mavrou K, Mackeogh T, McDonald R, Pettersson C, Scherer MJ (2018). Assistive technology and people: a position paper from the first global research, innovation and education on assistive technology (GREAT) summit. Disabil Rehabil Assist Technol.

[49] Selick A, Durbin J, Casson I, Lee J, Lunsky Y (2018). Barriers and facilitators to improving health care for adults with intellectual and developmental disabilities: what do staff tell us?. Health Promot Chronic Dis Prev Can.

[50] Chew KL, Iacono T, Tracy J (2009). Overcoming communication barriers - working with patients with intellectual disabilities. Aust Fam Physician.

[51] Pelleboer-Gunnink HA, Van Oorsouw WM, Van Weeghel J, Embregts PJ (2017). Mainstream health professionals' stigmatising attitudes towards people with intellectual disabilities: a systematic review. J Intellect Disabil Res.

[52] May ME, Kennedy CH (2017). Health and problem behavior among people with intellectual disabilities. Behav Analysis Practice.

[53] Fried-Oken M, Mooney A, Peters B (2015). Supporting communication for patients with neurodegenerative disease. NeuroRehabilitation.

[54] Vinales JJ (2013). Evaluation of Makaton in practice by children's nursing students. Nurs Child Young People.

[55] (2018). Guide to making information accessible for people with a learning disability. NHS England.

[56] (2020). How Makaton works. Makaton.

[57] Webb J, Stanton M (2009). Working with primary care practices to improve service delivery for people with learning disabilities – a pilot study. Br J Learn Disabil.

[58] Menzies R, Herron D, Scott L, Freeman R, Waller A (2013). Involving clinical staff in the design of a support tool improve dental communication for patients with intellectual disabilities. Proceedings of the 15th International ACM SIGACCESS Conference on Computers and Accessibility.

[59] Boström P, Broberg M (2018). Protection and restriction: a mixed-methods study of self-reported well-being among youth with intellectual disabilities. J Appl Res Intellect Disabil.

[60] Boström P, Eriksson E (2015). Design for self-reporting psychological health in children with intellectual disabilities. Proceedings of the 14th International Conference on Interaction Design and Children.

[61] Boström P, Johnels JA, Thorson M, Broberg M (2016). Subjective mental health, peer relations, family, and school environment in adolescents with intellectual developmental disorder: a first report of a new questionnaire administered on tablet PCs. J Ment Health Res Intel Disab.

