# Supplementary material for: Alternative and Augmentative Communication Technologies for Supporting Adults With Mild Intellectual Disabilities During Clinical Consultations: Scoping Review
Source: JMIR Rehabil Assist Technol. 2021 Jun 9;8(2):e19925. doi: 10.2196/19925 (PMC8235287; doi:10.2196/19925)
Supplement: Multimedia Appendix 1 [file rehab_v8i2e19925_app1.docx]

# Multimedia Appendix A: In-depth Overview of Identified Studies

**Table 1:** A summary of the studies identified by the authors as meeting the inclusion criteria during the data collection phase.

| Study | Aim | Intervention | Design | Population | Results |
| --- | --- | --- | --- | --- | --- |
| Jones & Kerr 1997 [1] | To evaluate an intervention that assists GPs in checking for common health conditions experienced by patients with ID. | A paper-based checklist containing information on appropriate health promotion activities i.e. whether they have been carried out in the past three years.  GPs also received educational packages on the best practices to employ when treating patients with ID. | Randomized controlled trial.  Information was collected on a range of variables including health promotion activities; consultation patterns; and patient well-being over a 6-month period. | 29 GPs from 6 practices identified 111 patients with ID to take part in the study.  56 of the participants with ID were male and 55 female (mean age 41 years). 28 had mild ID, 39 had severe and the severity of 44 was unknown.  30 participants had Down Syndrome. | No significant difference was observed in consultation patterns between the control and intervention groups across several factors including: location, nature and outcome.  No significant difference was observed across various health promotion activities.  The intervention identified some deficiencies in the frequency of recommended tests being carried out e.g. 15 of the 30 participants with Down Syndrome had not received a thyroid function test. |
| Dodd and Brunker 1999 [2] | To investigate whether it is possible to increase the health advocacy skills of people with ID. | An educational package designed to increase an individual with ID’s awareness of the human body, including common medical symptoms that may occur.  Computer generated images are also distributed to assist the patient in discussing site, type, severity and duration of pain, as well as basic medical symptoms such as sickness. | Pre and post intervention questionnaires on bodily functions and medical conditions. | Five men and five women aged between 31 and 46 years old. Their verbal skills varied widely – British Picture Vocabulary Test Scores ranged from 28 – 94.  No information on type or severity of ID was provided. | Improvements were observed in the participants’ knowledge of bodily processes and what to do when ill during 6-month follow-up period (although some reductions in retention occurred).  The highest degree of information retention was demonstrated by the participants who had visited their doctor and had used the communication aid resources within a clinical context. |
| Lennox et al. 2001 [3] | To pilot the Comprehensive Health Assessment Program and collect descriptive data regarding GPs views on the proposed strategies to improve the care of adults with ID.  As a by-product, the authors were able to determine the current health-related activities being conducted and the health problems detected as a result of such activities. | The Comprehensive Health Assessment Program (CHAP) is a booklet that contains the following resources:  A section to capture the medical history and screening activities of the patient with ID; A section that provides information to assist the GP in carrying out an assessment of the patient’s overall health; And a section to educate medical professionals on commonly missed or poorly managed conditions experienced by the ID population. | GPs were asked to recruit three patients with ID from their registers and utilise the CHAP booklet within consultations involving these patients over a 12-month period.  The intervention was then assessed via a self-evaluation form. | 15 GPs from 45 practices completed all components of the study.  38 patients with ID participated (21 females, 17 males) and were aged between 22 and 68 years old.  The type and severity of ID experienced by the participants was not described in depth, yet four participants were reported as having Down Syndrome. | Providing information on the medical history and screening activities of a patient can help medical professionals become aware of previously unknown conditions and keep up to date with health maintenance tasks.  The medical and screening information of the patient was considered a more valuable resource than the synopsis of the literature. |
| Lennox et al. 2004 [4] | To develop an advocacy tool that can be used by people with ID during a medical consultation. | The Ask It Health Diary which is a package containing a 5-year long diary and a brief advocacy educational session.  The diary contains the following sections:  Personal information about the patient such as communication preferences, health registrar details etc.  Resources to assist the patient in preparing for a consultation e.g. images of the human body and pain recording sheets.  Information on effective practices for clinical staff to use when treating patients with ID.  The medical history of the patient. | The intervention was piloted with two separate groups of individuals with ID over a 2-week period.  Feedback was then obtained during a phase of in-person or telephone interviews. | An advisory group met regularly throughout the project to provide feedback on the design of the intervention.  It consisted of: two2 people with ID, two support workers, two parent advocates,  two advocacy organisation representatives and an occupational therapist. Further feedback was sought from 22 professionals throughout the researchers’ network.  To determine the overall format and content of intervention, focus groups were carried out with eight people with ID, 85 support workers, three parents, two psychologists, one GP, one volunteer friend and a sister.  The final package was piloted by two groups: 19 parents of adults with intellectual disability who use a non-government support service; and seven people with intellectual disability who use a non-government accommodation service. | 66% of the participants interviewed felt that the diary would help them to become better advocates.  50% also felt that it would help them to improve their relationship with their GP.  GPs envisioned using the intervention with other populations. |
| Bell and Cameron 2008 [5] | To demonstrate the clinical advantages of employing the Talking Mats™ framework with patients with ID. | Talking Mats™ is a pictorial based communication aid. A visual scale is presented at the top of each mat, with the user required to give their views by placing relevant picture symbols under the appropriate section of the scale. | Talking Mats™ was used at two separate psychological consultations involving an individual ID.  The produced mats were then compared for similarities / differences and a report was created detailing the patient’s needs. This report was then approved by the patient and her social worker. | A woman in her 40s with mild ID. Her understanding of spoken, single words was similar to an individual aged between 14 years 9 months and 16 years 6 months. | Talking Mats™ took the pressure off the patient directly addressing her psychological concerns thus improving the quality and depth of information provided.  The information received was used to form a person-centred plan of action to improve the factors affecting the mental health of the patient.  Talking Mats™ can extend the use of therapies that rely heavily on verbal communication to those who ﬁnd verbal communication difﬁcult in a general sense, as well as a speciﬁc situational sense. |
| Lennox et al. 2010 [6] | To investigate whether 2 health assessment tools, the Ask health diary and the simpler CHAP tool, improved healthcare for people with ID. | Comprehensive Health Assessment Program - see details in row three (Lennox et al. 2001 [3])  Ask it diary – see details in  row 4 (Lennox et al. 2004 [4]) | A cluster randomised controlled trail was conducted with people with ID living in the Greater Brisbane area of Australia.  A 2 · 2 factorial design was used to examine the effects of the CHAP and Ask it health diary over a 12-month period. Health promotion, disease prevention and case finding activities were compared with those from the previous 12 months. | 272 individuals from 140 GPs agreed to participate in the baseline interviews.  Participating individuals were allocated into one of four factorial groups: usual care (77 participants), Ask only (57 participants), CHAP only (61 participants), or CHAP + Ask (77 participants).  89% (242) of the participants were involved in the follow-up stage.  The participants with ID’s mean age was 35 years, range 18-75. 107 had mild/moderate ID, 62 severe and 73 were unknown. | “Increased health promotion, disease prevention and case-ﬁnding activity were found in the intervention groups using the CHAP. It had a positive impact on Pneumococcus vaccination (OR 7.4; 95% CI: 1.5–37.1), hearing testing (4.5; 1.9–10.7), Hepatitis A vaccinations (5.4; 1.8–16.3), vision testing (3.4; 1.4–8.3), and weight measurement (3.1; 1.5–6.4). There were no strong changes in the measured outcomes in the group who used the Ask health diary alone. [6]”  The study period may have been too short to recognise the true benefits of the Ask it health diary. |
| Turk et al. 2010 [7] | To assess the impact of handheld health records (a form of patient passport) on GP consultations involving adults with ID. To determine if the proposed technology increases the health knowledge of people with ID and / or their carers. | The personal health passport consists of 50 double-sided pages separated into three sections. The first is a brief introduction, with the second encapsulating chapters on the health of the individual e.g. medication being taken, mental health symptoms etc. The final section contains information on ID for use by carers or medical professionals. | Randomised Controlled Trial in which consenting GP practices, containing patients with ID on their registrars, were randomly allocated to the control or intervention group.  Primary outcome measures were obtained from GP records and included: basic demographic information; degree and type of disability; the completion of the OK Health Checklist – considered to be the most comprehensive health assessment tool; a newly devised Knowledge of Health Problems and Terminology Checklist (KHPT) to measure the number and type of health needs known by the patient with ID and/or their carer; the Client Services Receipt Inventory to measure the number of GP visits; and questions relating to the satisfaction of primary care consultation including communication with their GP.  These measures were repeated during the follow-up interviews (conducted a minimum of 12 months after the commencement of the study), with additional questions being presented on the use of the health record where appropriate. | Forty GP practices were randomised to the intervention or control group. 201 people with ID were interviewed at baseline and 163 followed up after 12 months intervention. People with ID and carers were also employed as research interviewers.  Baseline interviews were completed for 102 participants with ID in the PHP intervention group and 99 in the control group. 89 carers out of the 168 involved in baseline interviews with people with ID knew the cause of the participants disability. This included:  Down syndrome – 27 (16%), autism spectrum disorder – 21 (12.5%), cerebral palsy – 16 (9.5%), another syndrome – 12 (7.1%), and other congenital factors, peri-natal birth problems or epilepsy – 11 (6.7%). | No signiﬁcant outcomes were achieved by the intervention.  Annual consultation rates in the intervention and control groups at baseline were low (2.3 and 2.6 visits respectively). A slightly greater increase occurred over the year in the intervention group 0.6 ()0.4 to 1.6) visits per year compared with controls. People with ID in control group reported more health problems at follow-up 0.9 (0.0 to 1.8). People with ID liked their health record (92%) but only 63% reported usage. |
| Brodrick et al. 2011 [8] | To improve the health experiences of people with ID using services in East Cheshire via the development of a one-page patient passport. | The passport contains personal information about the patient that will assist medical professionals in performing person centred care. It is split into four sections in order of priority: medical information; communication needs; support needs; and environmental needs.  A personal information page (on the reverse) also encapsulates information deemed important by administrative and clinical staff from the emergency and outpatient departments. | A pilot study was carried out in October 2009 in Macclesfield District General Hospital and two independent service providers: David Lewis and The Rossendale Trust.  Residential managers were trained to use the passports, who then introduced them to frontline staff. Circa 150 passports were produced over the trial period, although no additional information was provided on the manner in which they were developed.  The quality of the passports was reviewed by the researchers during the pilot stage and initial feedback was gained from discussion groups held by working group members and staff. | “A working group was established with representation from East Cheshire NHS Trust; Cheshire and Wirral Partnership NHS Foundation Trust; East Cheshire Advocacy (an organisation that provides professional and volunteer advocacy support to people with learning disabilities); and the local independent sector, including David Lewis (a registered charity providing education, therapy, support and life skills development to people with epilepsy and complex learning disabilities) and The Rossendale Trust (an organisation that supports people with learning and physical disabilities).” [8]  Working group members initially sought guidance from administrative and clinical staff at Macclesfield District General Hospital’s emergency and outpatient departments to ascertain the essential information required when a person with learning disabilities accesses their services  A pilot study was carried out in October 2009 in Macclesfield District General Hospital and two independent service providers: David Lewis and The Rossendale Trust.  Residential managers were trained to use the passports, who then introduced them to frontline staff. Circa 150 passports were produced over the trial period, although no additional information was provided on the manner in which they were developed. | The pilot study shows that the passport has the potential to improve the quality of support being provided to patients with ID on admission to and during their stay in hospital.  The initial quality of the passports was extremely variable, with some not containing the information required to be effective. This improved when additional support and training was provided, as well as example templates.  “Staff found the patient passport to be a useful document because it enabled them to provide pertinent, person-centred information about the service user in a simple, clear format. This saved time on admission.” [8] |
| Bell 2012 [9] | To evaluate a hospital passport tool and examine its effects on communication between patients with ID and staff. | The passport is an A4 sized, coloured booklet that includes red, amber and green sections detailing personal information that may assist in providing person-centred care.  There is also a Vital Information Sheet to clearly emphasise potentially lifesaving information, for example, if the person has dysphagia (swallowing problems) and how to manage this safely. | Semi-structured interviews involving family members and health and social care staff who had supported an individual with ID when using the passport. A focus group that involved people with ID discussing their experiences of staying in hospital. A reﬂexive journal analysis that included the researcher’s views on a recent example of the passport in practice.  The 3 separate studies allowed for triangulation. | “12 participants including health, hospital and social care workers as well as family carers, were asked a series of ﬁve questions about their experiences, both positive and negative, of using the Trafﬁc Light Hospital Assessment. Additionally, eight participants, all of whom were adults with learning disabilities attending a self-advocacy group, took part in a focus group discussion about their experiences of going into hospital.” [9] | The passport was a useful resource in improving communication and the continuity of care across multiple agencies involved in the treatment of patients with ID.  Passports can help improve communication between patients, carers, and medical professionals, which can have a direct impact on the quality of care being provided. |
| Heifetz and Lunsky 2018 [10] | To evaluate the use of health passport communication tools by people with ID in psychiatric and general emergency departments throughout Ontario, Canada. | Patient passports were locally tailored and implemented throughout three regions of Ontario. All contained information on: the patient’s medical history; and their baseline behaviours e.g. communication preferences, signs of distress, behavioural triggers etc.  Nevertheless, each regions’ chosen passport differed in terms of size (wallet sized vs. one full double-sided page vs. four pages) and in aesthetics e.g. plain written information vs. picture based. | The intervention was evaluated through interviews with those responsible for implementing the passports in each region, as well as interviews with stakeholders and surveys.  Each of these stages were carried out between 6 and 12 months after the implementation of the passport. | 28 questionnaires and 18 interviews with stakeholders (e.g., hospital staﬀ, community agency representatives, families) were completed throughout the regions.  The questionnaire was completed by: three individual clients / patients (11%); seven family members (25%); and 18 support staﬀ / paid caregivers (64%).  82% of questionnaire participants failed to use the passport within a clinical setting.  18 semi-structured interviews were conducted over the telephone with: hospital clinical staﬀ, community health and ID service providers, and community-based health care case coordinators. | 21 participants who completed the questionnaire (75%) reported that the tool “deﬁnitely” provides background information about the patient, and ~50% felt that the tool “deﬁnitely” makes the patient more comfortable.  Benefits were also discussed in regards to: helping the caregiver feel more involved and respected (n = 13, 65%), helping hospital staﬀ to adapt their care approach (n =13, 65%), helping in improving communication with hospital staﬀ (n= 16, 80%), and helping make decisions with better information (n = 16, 80%).  The levels of adoption of the passport varied by region. Strong leadership or “medical champions” are required to increase use by front-line care staff. |
| Gibson et al. 2018 [11] | To demonstrate the potential use of mobile technologies to meet the communication needs of patients with mild ID during primary care consultations. | A hi-tech, clinical alternative and augmentative communication tablet application.  The application intends to extract medical information from the patient, using an accessible questionnaire, prior to the consultation.  As such, the primary symptoms the patient is experiencing may be available - in an accessible format - to all stakeholders thus potentially improving communication. | Requirements gathering interviews were carried out with ten experts in ID, which included: two GPs; three Governmental advisor’s involved in the development of Scotland’s national ID strategy; four academics in the fields of social work, cognitive psychology, inclusive education, and aging, fragility and dementia; and a full-time support worker.  A hi-fidelity prototype was then developed using the identified requirements and subsequently presented to a subset of these experts during a usability study. | 10 experts with ID were involved in the requirements gathering interviews including: two GPs; three Governmental advisor’s involved in the development of Scotland’s national ID strategy; four academics in the fields of social work, cognitive psychology, inclusive education, and aging, fragility and dementia; and a full-time support worker.  Four of these ten experts then participated in a usability study to determine if there were major accessibility issues in the developed prototype prior to introducing additional participants with mild ID. | The proposed application has the potential to alleviate those issues that arise from cuts in funding to support workers by increasing the health advocacy skills of patients with mild ID.  The GPs interviewed were open to including AAC technologies within the consultation process despite their lack of experience in using such technologies.  The application has the potential to highlight the conditions commonly overshadowed by professionals undereducated on the health needs of people with ID.  Providing medical information in advance of the consultation can help reduce debilitating time constraints. It also enables medical professionals to concentrate on areas of interest for longer. |
| Gibson et al. (2019) [12] | To assess the feasibility of utilising tablet applications to increase the quality of communication between GPs and patients with mild ID. | A hi-tech, clinical, alternative and augmentative communication tablet application.  The application intends to extract medical information from the patient, using an accessible questionnaire, prior to the consultation.  As such, the primary symptoms the patient is experiencing may be available - in an accessible format - to all stakeholders thus potentially improving communication. | Requirements gathering interviews were carried out with ten experts in ID.  A hi-fidelity prototype was then developed using the identified requirements and subsequently presented to a subset of these experts during a usability study. | 10 experts with ID were involved in the requirements gathering interviews including: two GPs; three Governmental advisor’s involved in the development of Scotland’s national ID strategy; four academics in the fields of social work, cognitive psychology, inclusive education, and aging, fragility and dementia; and a full-time support worker.  4 of these 10 experts then participated in a usability study to determine if there were major accessibility issues in the developed prototype prior to introducing additional participants with mild ID. | Three primary advantages of the proposed application were discussed by the experts: (1) promoting communication by presenting medical information in a manner that is accessible to all stakeholders; (2) alleviating time constraints by collecting medical information prior to the consultation – this enables medical professionals to concentrate on areas of interest for longer; and (3) highlighting medical conditions that are commonly overshadowed by practitioners. |
| Gibson et al. (2019a) [13] | To investigate the use of AAC applications to promote the exchange of information between GPs and patients with mild ID. This was achieved by identifying initial requirements that cater to the needs of patients with ID. | A hi-tech, clinical alternative and augmentative communication tablet application.  The application intends to extract medical information from the patient, using an accessible questionnaire, prior to the consultation.  As such, the primary symptoms the patient is experiencing may be available, in an accessible format, to all stakeholders thus potentially improving communication. | 2 focus groups were carried out with 12 experts in ID to: first determine the accessibility of a future user-centred design workshop for adults with mild ID; and second, identify the experts views on how the application should look and function.  The activities included: a focus group to explore the communication challenges experienced by patients with mild ID, as well as how technology can be used to overcome these challenges; an image board exercise to identify factors that constitute effective medical images; a paper prototyping process to identify the features to be included in the app; and a post-task walkthrough of a similar application. | Focus group one was conducted in the city of Glasgow, Scotland and included: three academics in the health and wellbeing of people with ID; two employees of an advocacy charity, one of which had mild ID; a former ID nurse who is now manager of a support centre for people with ID; and a digital inclusion officer.  Focus group two was conducted in the city of Dundee, Scotland and consisted of: three community ID nurses; one employee of an advocacy charity; and one employment support officer. | Similar advantages to the above two studies were discussed by the experts involved.  Additionally, the experts in focus group two discussed the importance of including personal information on aspects such as the communication needs of the patient. This will assist medical professionals in adjusting their consultation methods to improve the quality of care provided. |
| Raemy and Paignon (2019) [14] | To demonstrate the extent of adjustments required to provide high-quality care to patients with ID in an acute care setting in Western Switzerland.  These adjustments focused on: patient care pathways; the education of professionals about ID; the employment of communication aids to standardise care; the accessibility of the hospital’s environment; and the employment of specialised ID health staff. | To improve communication throughout different sections of the hospital, an emergency admission sheet (similar to a patient passport) was developed.  The admission sheet is an A4 sized, double-sided document that includes information deemed crucial to the care of the patient including: their disability; challenging behaviours and the presence of additional impairments;  legal representation; capacity to consent; and communication abilities such as how they express pain, comfort, discomfort and anger.  This admission sheet is standardised in the electronic patient data system to ensure it is available to all health professionals involved in the patient’s care. | In 2012, 60 working group sessions took place to determine the most common health needs of the ID population, the barriers to effective care, and the  prioritisation of adjustments that may be made to improve this care.  Multidisciplinary teams consisting of nurses, medical doctors, physiotherapists, senior nurses, social workers, representatives of families, associations, architects, representatives of the main supported residential accommodations and lecturers from the Nursing department of the University of Applied Sciences were then set up to address the most important issues identified in the working group sessions.  The multidisciplinary teams were responsible for improving 4 aspects: Patient care pathways and best practice; communication; human resources; and environment.  Annual reviews then occurred between 2012 and 2017 to share the progress / challenges that occurred during the implementation of the reasonable adjustments.  An overall evaluation of the project started in 2016 and is still on-going. Patient satisfaction  questionnaires regarding the efficiency of the adjustments made and the overall quality of care received is the primary means of evaluation being collected. | Multidisciplinary teams consisting of nurses, medical doctors, physiotherapists, senior nurses, social workers, representatives of families, associations, architects, representatives of the main supported residential accommodations and lecturers from the Nursing department of the University of Applied Sciences were then set up to address the most important issues identified in the working group sessions.  An overall evaluation of the project started in 2016 and is still on-going. Patient satisfaction  questionnaires regarding the efficiency of the adjustments made and the overall quality of care received. | The emergency admission sheet has been filled in by almost every registered individual with ID in Geneva. It is systematically used throughout the hospital and has resulted in improved communication. Nevertheless, there is an estimated 10% of people with ID who have not been diagnosed meaning they cannot benefit from the admission sheet.  An ID-physician and ID-nurse have also been employed to assist medical professionals in improving the care being provided to patients with ID and to educate them on best practices etc. This has resulted in improved care being administered to approximately 100 patients over the year 2016, over 400 in 2017 and 517 in 2018. |
| Chinn (2019) [15] | To determine the impact easy read information has on the literacy events present in consultations involving patients with ID. | Easy Read is the term used to describe written resources that have been adapted to cater to the needs of people with ID. They typically include short, jargon free sentences supported via the use of immediately identifiable imagery.  One such resource used within the study was a leaflet on blood tests that both described and highlighted the steps involved in the procedure. | 32 ID health check procedures involving primary care clinicians who had access to easy read resources were video recorded, as well as 9 health appointments with ID nurses.  These recordings were then analysed using conversation analysis to examine the interactional micro‐practices that frame literacy events involving easy read texts. Using conversational analysis ensures that the data is captured from naturally occurring interactions as opposed to post hoc interviews or surveys.  Reflective interviews were also conducted with nine of the health staff and nine of the patients. They were required to watch the recordings of the consultation and elaborate on the actions performed during areas of interest. | The full data set comprised video and audio recordings of 32 ID health checks conducted by GP staff and 9 video and audio recordings of health consultations between people with ID and specialist ID nurses. Four patients attended health checks by themselves, and others were accompanied by supporters who were either family members or people in paid support roles.  Recruiting staff involved in performing the health checks of people with ID meant that the presence of the target population was guaranteed.  The rationale behind recruiting community ID nurses was that they might use different communicative strategies based on their more extensive experiences with patients with ID compared to the primary care staff, and also that they were more likely to routinely use easy read resources. | Easy Read health information was visible in only 7 (22%) of the primary care health checks (though not always shared with the patients). Easy Read health information was used in sequences where clinicians offered unsolicited health advice and were met with degrees of resistance from patients, though its potential for shared decision making was also evident.  The specialist intellectual disability nurses were more likely to use Easy Read formatted resources of all kinds in their recorded interactions. Such resources were part of the interaction with patients with ID in four of the nine recordings made. The easy read texts were also more varied than those evident in the GP settings. |

## References

1. Jones, R.G., Kerr, M.P.: A randomized control trial of an opportunistic health screening tool in primary care for people with intellectual disability. J Intellect Disabil Res. 41 ( Pt 5), 409–415 (1997)

2. Dodd, K., Brunker, J.: ‘Feeling Poorly’: Report of a Pilot Study Aimed to Increase the Ability of People with Learning Disabilities to Understand and Communicate About Physical Illness. British Journal of Learning Disabilities. 27, 10–15 (1999). https://doi.org/10.1111/j.1468-3156.1999.tb00076.x

3. Lennox, N.G., Green, M., Diggens, J., Ugoni, A.: Audit and comprehensive health assessment programme in the primary healthcare of adults with intellectual disability: a pilot study. J Intellect Disabil Res. 45, 226–232 (2001). https://doi.org/10.1046/j.1365-2788.2001.00303.x

4. Lennox, N., Taylor, M., Rey-Conde, T., Bain, C., Boyle, F.M., Purdie, D.M.: ask for it: development of a health advocacy intervention for adults with intellectual disability and their general practitioners. Health Promot Int. 19, 167–175 (2004). https://doi.org/10.1093/heapro/dah204

5. Bell, D.M., Cameron, L.: From Dare I say … ? to I dare say: a case example illustrating the extension of the use of Talking Mats to people with learning disabilities who are able to speak well but unwilling to do so. British Journal of Learning Disabilities. 36, 122–127 (2008). https://doi.org/10.1111/j.1468-3156.2007.00475.x

6. Lennox, N., Bain, C., Rey‐Conde, T., Taylor, M., Boyle, F.M., Purdie, D.M., Ware, R.S.: Cluster Randomized-Controlled Trial of Interventions to Improve Health for Adults with Intellectual Disability Who Live in Private Dwellings. Journal of Applied Research in Intellectual Disabilities. 23, 303–311 (2010). https://doi.org/10.1111/j.1468-3148.2009.00533.x

7. Turk, V., Burchell, S., Burrha, S., Corney, R., Elliott, S., Kerry, S., Molloy, C., Painter, K.: An Evaluation of the Implementation of Hand Held Health Records with Adults with Learning Disabilities: A Cluster Randomized Controlled Trial. Journal of Applied Research in Intellectual Disabilities. 23, 100–111 (2010). https://doi.org/10.1111/j.1468-3148.2009.00518.x

8. Brodrick, D., Lewis, D., Worth, A., Marland, A.: One-page patient passport for people with learning disabilities. Nurs Stand. 25, 35–40 (2011). https://doi.org/10.7748/ns2011.07.25.47.35.c8644

9. Bell, R.: Does he have sugar in his tea? Communication between people with learning disabilities, their carers and hospital staff. Tizard Learning Disability Review. 17, 57–63 (2012). https://doi.org/10.1108/13595471211218712

10. Heifetz, M., Lunsky, Y.: Implementation and evaluation of health passport communication tools in emergency departments. Res Dev Disabil. 72, 23–32 (2018). https://doi.org/10.1016/j.ridd.2017.10.010

11. Gibson, R.C., Bouamrane, M.-M., Dunlop, M.: Mobile support for adults with mild learning disabilities during clinical consultations. In: Proceedings of the 20th International Conference on Human-Computer Interaction with Mobile Devices and Services - MobileHCI ’18. pp. 1–8. ACM Press, Barcelona, Spain (2018)

12. Gibson, R.C., Bouamrane, M.-M., Dunlop, M.: Design Requirements for a Digital Aid to Support Adults With Mild Learning Disabilities During Clinical Consultations: Qualitative Study With Experts. JMIR Rehabilitation and Assistive Technologies. 6, e10449 (2019). https://doi.org/10.2196/10449

13. Gibson, R.C., Bouamrane, M.-M., Dunlop, M.: Experts Views on the Use of Mobile Devices to Support Patients with Mild Learning Disabilities During Clinical Consultations. Stud Health Technol Inform. 264, 1199–1203 (2019). https://doi.org/10.3233/SHTI190416

14. Lalive d’Epinay Raemy, S., Paignon, A.: Providing equity of care for patients with intellectual and developmental disabilities in Western Switzerland: a descriptive intervention in a University Hospital. Int J Equity Health. 18, 46 (2019). https://doi.org/10.1186/s12939-019-0948-8

15. Chinn, D.: An empirical examination of the use of Easy Read health information in health consultations involving patients with intellectual disabilities. J Appl Res Intellect Disabil. (2019). https://doi.org/10.1111/jar.12657
